# *In situ* polymerization and electrical conductivity of polypyrrole/cellulose nanocomposites using Schweizer's reagent†

**DOI:** 10.1039/d2ra04320c

**Published:** 2022-08-09

**Authors:** Stephanie A. Fraser, Werner E. van Zyl

**Affiliations:** School of Chemistry and Physics, University of KwaZulu-Natal, Westville Campus Durban 4000 South Africa vanzylw@ukzn.ac.za

## Abstract

Cellulose-based composites have attracted interest given the shift towards ‘green’ materials, but achieving uniform dispersions of cellulose in polymer matrices and/or enhancement of interfacial interactions between components remains challenging. Herein we report the preparation of polypyrrole/cellulose nanocomposites in [Cu(NH_3_)_4_(H_2_O)_2_](OH)_2_ (Schweizer's reagent/cuoxam)-based reaction media *via in situ* polymerization. The effect of cellulose template morphology and reaction media on the microstructure, electrical conductivity, and surface wettability was studied. Aqueous reaction media favored the formation of a uniform polypyrrole coating encapsulating the cellulose fibers; concentrated cuoxam solutions promoted inhomogeneity and exhibited a progressive decline in conductivity. The maximum conductivity attained was 3.08 S cm^−1^ from a bacterial cellulose-templated composite prepared in aqueous reaction media and afforded an approximately threefold increase in conductivity when compared with pure PPy at 1.14 S cm^−1^. Generally, the composites resembled wetting surfaces – with highly concentrated cuoxam solutions yielding improved hydrophilicity, while substitution of bacterial cellulose with nanocrystalline cellulose engendered a shift towards hydrophobicity. Most composites displayed a contact angle of less than 90° suggesting PPy/cellulose composites tended towards hydrophilic behavior. This study highlights investigations into the viability of cellulose solvents as a facile means to control the structure and performance of *in situ* functionalized cellulose nanocomposites.

## Abstract 2

The *in situ* polymerization of polypyrrole/cellulose nanocomposites using Schweizer's reagent is demonstrated and measured against a number of structural, electrical, and colligative properties.

## Graphical abstract

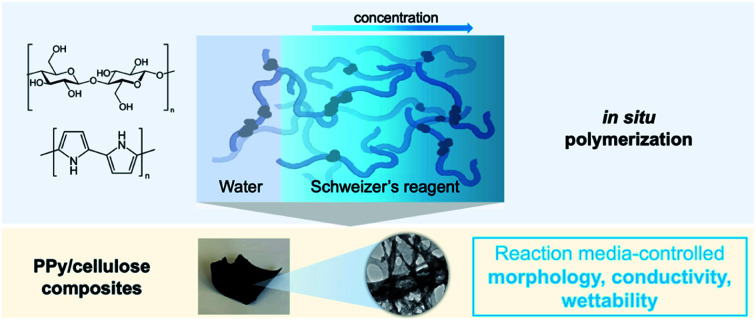

## Introduction

Intrinsically conducting polymers (ICPs) have attracted widespread interest due to their unique optical properties, exceptional electroconductivity, electrochemical activity, and biocompatibility.^1^ Polypyrrole (PPy) is a particularly appealing candidate on account of its non-toxicity, resistance to oxidation, and environmental stability.^2,3^ Application of this polymer, however, is limited by poor processability and inadequate mechanical properties.^2^ Currently, there is growing interest in the hybridization of PPy with other materials to generate functional nanocomposites.^4–7^ Coating a support matrix with ICPs is a ubiquitous approach used to prepare flexible ICP films or membranes.^8,9^ Deposition of the polymer on templating fibers (*e.g.* cotton,^10,11^ silk,^12^ polyamide,^13^ polyester^14^) to form conductive fibrous matrices has primarily been achieved through *in situ* oxidation.^2^ Moreover, natural fibers – particularly cellulose – offer a ‘greener’ alternative to conventional templating agents.^15^

Cellulose is the most abundant biopolymer on Earth and comprises β-1,4-linked anhydroglucose units.^16^ Consolidation of the unique properties of cellulose (biocompatibility, biodegradability, polyfunctionality and hydrophilicity) with the aforementioned electrical properties of PPy can yield synergistic effects.^17^ Bacterial cellulose (BC) is a specific type of cellulose produced *via* bottom-up synthesis. A variety of *Acetobacter* and *Gluconobacter* bacteria orchestrate the conversion of biochemically activated glucose monomers into a 3-D network of ribbon-like BC fibers with a cross-sectional width of 20–100 nm.^18–22^ BC serves as a more sustainable alternative to plant cellulose because, unlike cellulosic biomass which undergoes extensive treatment and processing (to remove hemicelluloses and lignin), BC can be easily biosynthesized in diverse media to meet demand without post-treatment.^23–25^ As a consequence of its unique architecture, BC possesses high purity, crystallinity and water-holding capacity, as well as a high degree of polymerisation.^16,23,26^ Thus, BC also boasts much of the benefits associated with nanocrystalline cellulose (NC), whilst avoiding time-consuming and costly processing associated with finer nanostructures. In the context of this study focusing on conductive composites, BC makes for an ideal scaffold. Generally, BC fibers possess a relatively high aspect ratio coupled with a 3-D network structure.^27^ Hence, conductive BC composites benefit from a low percolation threshold, such that less material is required to achieve substantial conductivity which is hugely advantageous from both an economic and environmental perspective. Thus, we narrow our focus to PPy/BC nanocomposites. Several studies have also demonstrated the polymerization of pyrrole in a variety of reaction media, including ionic liquids, protic and aprotic solvents, and polymeric solutions.^28–30^ Additionally, various solvents, including chloroform, methanol and tetrahydrofuran, facilitate cellulose swelling to a greater extent than water. Implementation of these solvents in the preparation of PPy/cellulose composites is reported to improve homogeneity and conductivity.^31^

In this study we report a novel approach for the synthesis of polypyrrole/cellulose nanocomposites, using cuoxam-based solvent systems as reaction media. This solvent – also referred to as Schweizer's reagent – bears the chemical formula [Cu(NH_3_)_4_(H_2_O)_2_](OH)_2_. It has been demonstrated that this ammoniacal solution of copper(ii) hydroxide can completely dissolve cellulose (and other polyols) under highly alkaline conditions.^32,33^ In previous work we have successfully utilized this solvent for the *in situ* functionalization of cellulose containing a luminescent inorganic cluster, and we also demonstrated the formation of Kombucha-based bacterial nanocellulose embedded in a polypyrrole/PVA composite.^34,35^ Due to the solubility of the template (*i.e.* cellulose) in cuoxam, it was anticipated that the resultant composites, prepared *via* a classic ‘bottom-up’ synthesis approach, would exhibit improved homogeneity – and hence enhanced electroconductivity relative to analogous composites prepared in aqueous media. Furthermore, two distinct templating agents, namely (i) bacterial cellulose (BC) and (ii) nanocrystalline cellulose (NC), were implemented in the preparation of these composite materials. The unique structural arrangement of these materials in solution was expected to cause microstructural differences between the resultant PPy/BC and PPy/NC composites. It was predicted that this would also influence electroconductivity and surface wettability. The effect of reaction duration on these properties was also investigated.

## Experimental section

### Materials

All commercially available chemicals were reagent grade and used without further purification. Pyrrole (C_4_H_5_N, ≥98%, *M*_w_ = 67.09 g mol^−1^) and copper(ii) sulfate pentahydrate (CuSO_4_·5H_2_O, ≥98.0%, *M*_w_ = 249.68 g mol^−1^) were obtained from Sigma-Aldrich Co. Ltd. Iron(iii) chloride hexahydrate (FeCl_3_·6H_2_O, ≥99%, *M*_w_ = 381.37 g mol^−1^) was obtained from ACE (Pty) Ltd. Potassium hydroxide (KOH, ≥88.7%, *M*_w_ = 56.11 g mol^−1^) was obtained from Set Point Laboratories. Hydrochloric acid (HCl, 30–34%, *M*_w_ = 36.46%) was purchased from Merck (Pty) Ltd. Ammonia gas (NH_3_) was purchased from Afrox (South Africa). Deionized water was used in the preparation of all aqueous solutions. Suspensions of bacterial cellulose and nanocrystalline cellulose were prepared as described in the ESI.†

### Powder X-ray diffraction

XRD measurements were obtained using a Bruker multi-purpose X-ray diffractometer D8-Avance operated in a continuous θ–θ scan in locked coupled mode with copper radiation. The sample was mounted in the center of the sample holder on a glass slide and levelled up to the correct height. The measurements were obtained at a typical step size of 0.034° in 2θ. A position-sensitive detector, Lyn-Eye, was used to record diffraction data at a speed of 0.5 s/step. The data was processed using EVA software from Bruker.

### Fourier transform infrared spectroscopy

Spectra were recorded on a PerkinElmer Spectrum 100 FT-IR spectrometer fitted with a universal attenuated total reflectance sampling accessory. The OMNIC™ 7.0 Professional Software Suite (Thermo Scientific) was used to process the raw spectra.

### Transmission electron microscopy

A tiny fragment of each solid composite was sonicated in water to produce a suspension. A small volume of the suspension was transferred onto a copper grid and left to dry for approximately ten minutes under a heat lamp. All samples were stained with a drop of uranyl acetate (1% w/v solution) to enable visualization of cellulosic material. TEM images were captured using a JEOL 2100 (Japan) high-resolution transmission electron microscope (HRTEM) operated at 20 kV. Composite thickness measurements were made using a digital Vernier caliper instrument with a resolution of 0.01 mm. The results are reported in Table S1.†

### Synthesis of polypyrrole/cellulose composites

A series of polypyrrole/cellulose composites were prepared in two types of reaction media: (i) H_2_O (A) and (ii) cuoxam solutions of varying concentration (B, C, D). Additionally, the composites incorporated one of two cellulose starting materials: (i) bacterial cellulose or (ii) nanocrystalline cellulose, denoted BC and NC, respectively. Reaction duration was fixed at 50 minutes or 20 hours, denoted 1 and 2, respectively. Preparation and characterization of the cellulosic materials is described in the ESI.† The various chemical compositions and reaction conditions implemented for the synthesis of these materials are recorded in Table 1. The modified procedure outlined below is adapted from the methodology reported by Wang *et al.*^36^

**Table 1:** Chemical composition and reaction conditions for the preparation of the composites

Sample label	Reaction time^a^	Feeding mass ratio of cellulose: CuSO_4_·5H_2_O	Cellulose type^b^
BC-A1	50	—	BC
BC-B1	50	1 0.5	BC
BC-C1	50	1 3	BC
BC-D1	50	1 20	BC
BC-A2	20	—	BC
BC-B2	20	1 0.5	BC
BC-C2	20	1 3	BC
BC-D2	20	1 20	BC
NC-A2	20	—	NC
NC-C2	20	1 3	NC

a The reaction duration was either 50 minutes or 20 hours.

b BC and NC denote bacterial and nanocrystalline cellulose, respectively.

### Preparation of the oxidant/dopant solution

A solution was prepared by combining FeCl_3_·6H_2_O (7 mmol, 0.45 M) and HCl (16.8 mmol, 1 M), which function as the oxidant and the dopant, respectively.

### Synthesis of composites in H_2_O (A)

A mass of BC (1) or NC (2) (50 mg) was diluted to a final volume of 150 mL. The aqueous cellulose suspension was stirred for 20 minutes to facilitate dispersion of the cellulose. Thereafter, a volume of pyrrole (500 μL) was added all at once and stirring was continued for a further 20 minutes. The reaction vessel was then transferred to an ice bath. Once cooled to 0 °C, the oxidant/dopant solution was added dropwise under vigorous stirring. The reaction was allowed to proceed under dark conditions for a duration of 50 minutes (X) or 20 hours (Y) while maintaining a constant temperature. The resulting grey/black suspension was filtered *in vacuo* and washed with water until the filtrate became colorless. This was followed by further washing with aliquots (10.0 mL) of acetone and HCl (1 M). The composites were then transferred to a desiccator to dry. Thereafter, the composites were separated from the filter paper, transferred to glass vials, and stored in a desiccator at room temperature.

### Synthesis of composites in cuoxam solutions (B–D)

The feeding mass ratios of cellulose to CuSO_4_·5H_2_O were 1 : 0.5, 1 : 3 and 1 : 20 and the corresponding composites were labelled B, C and D, respectively. A typical procedure for the preparation of samples labelled B involved the dissolution of CuSO_4_·5H_2_O (25 mg, 0.10 mol) in water. An aqueous KOH solution (0.23 g, 4.1 mmol) was added to the CuSO_4_·5H_2_O solution under vigorous stirring to form a pale blue precipitate (*i.e.*, Cu(OH)_2_). Ammonia gas was bubbled through the precipitate under stirring, resulting in a dark blue solution (*i.e.*, [Cu(NH_3_)_4_(H_2_O)_2_](OH)_2_ or Schweizer's reagent). A mass of BC (1) or NC (2) (50 mg) was added to the cuoxam solution and diluted to a final volume of 150 mL. The cellulose/cuoxam solution was stirred for 20 minutes to facilitate dissolution. Thereafter, a volume of pyrrole (500 μL) was added all at once, and stirring was continued for a further 20 minutes. The reaction vessel was then transferred to an ice bath. Once cooled to 0 °C, the oxidant/dopant solution was added dropwise under vigorous stirring. The reaction was allowed to proceed under dark conditions for a duration of 50 minutes (X) or 20 hours (Y) while maintaining a constant temperature. The resulting turbid brown solution was poured into an excess of 32% HCl solution, resulting in the gradual precipitation of black fibrous material in a clear yellow solution. The black solid was filtered *in vacuo* and washed with water until the filtrate became colorless. This was followed by further washing with aliquots (10.0 mL) of acetone and HCl (1 M). The composites were then transferred to a desiccator to dry. Thereafter, the composites were separated from the filter paper, transferred to glass vials, and stored in a desiccator at room temperature.

### Sheet resistance measurement

Sheet resistance measurements were made using a four-point probe (FPP) head (Jandel Cylindrical Four Point Probe) mounted on a height-adjustable stand. The probe was connected to a source measure unit instrument (Keithley 2450 SourceMeter), and measurements for each composite were recorded at an operating current of 1 μA. Multiple readings for each sample were obtained by adjusting the position of the probe across the surface of the film. The average of these results was recorded.

### Contact angle measurement

The contact angle measurements were obtained by the following procedure. The composite film was fixed to a flat surface. A drop (10 μL) of double distilled water was placed on the surface of the film being tested. After 10 seconds, a digital photograph of the interface between the drop and the specimen surface was captured using an Apple iPhone X mobile phone with a 12 MP (megapixel) wide-angle camera fitted with a detachable magnification lens. The photographs were digitally processed to meet software requirements. The contact angle was determined using ImageJ software in conjunction with the drop analysis software plug-in developed by Stalder *et al.*^37^ For each specimen, several measurements were made at several sites on the sample surface to obtain an average, reported as the final result.

## Results and discussion

### Synthesis of polypyrrole/cellulose composites

**Scheme 1:**
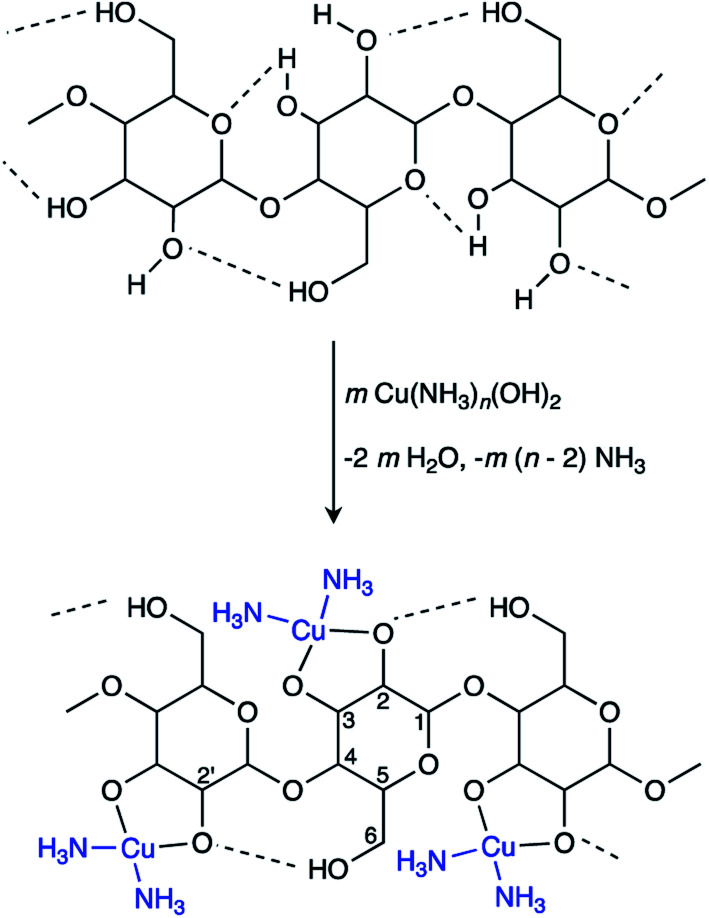
Mechanism for the dissolution of cellulose in cuoxam.

**Scheme 2:**
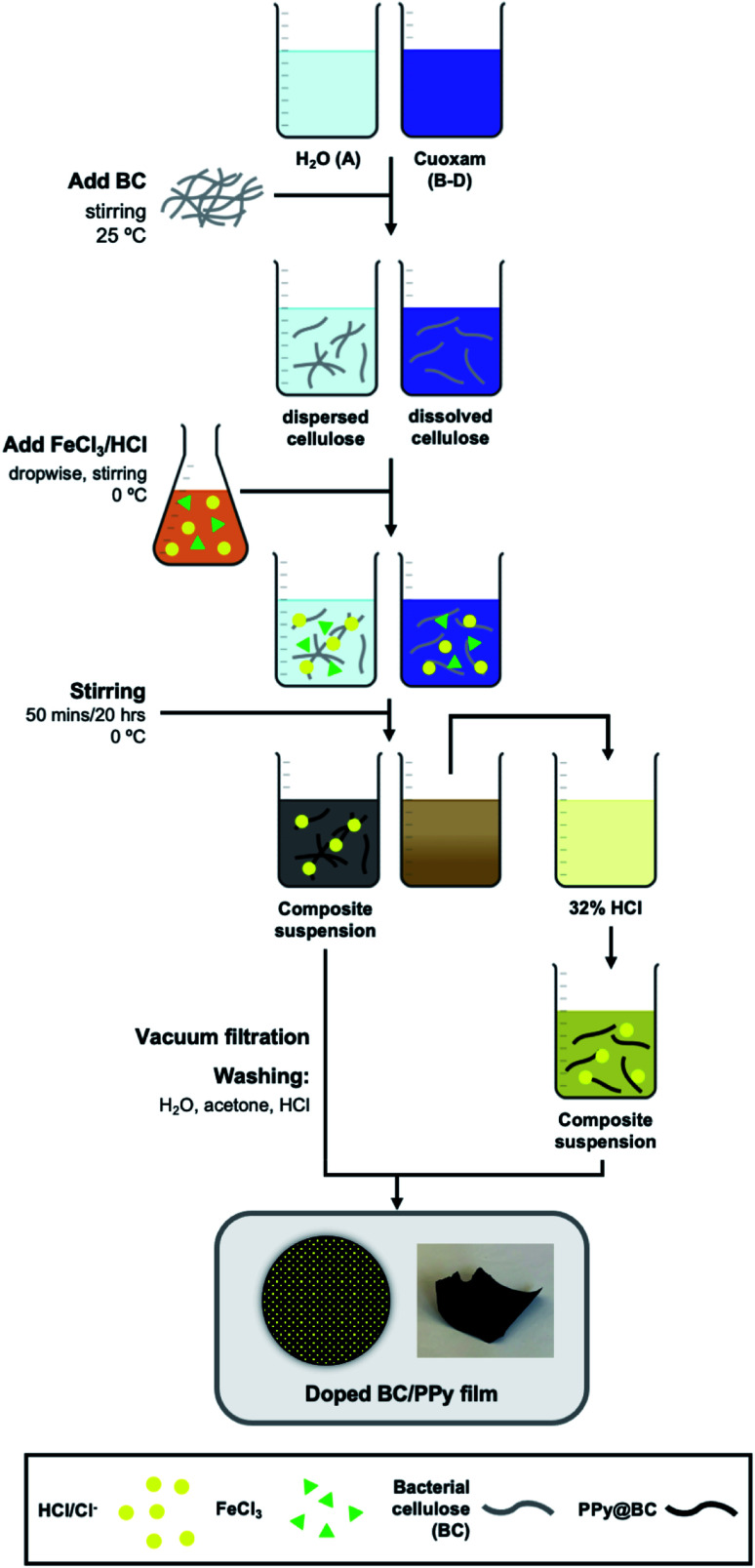
Methodology for the preparation of PPy/BC composites.

The proposed mechanisms for PPy chain-formation are described in Scheme S1.† Regardless of the true polymerization mechanism, it is evident that positively charged intermediates are involved in PPy synthesis. Therefore, it was hypothesized that templating agents with anionic moieties would improve the binding of the final PPy product to the template through the presumed coulombic attraction of intermediates during polymerization. This, along with the ability of cuoxam to uniformly disperse the cellulose template, was the rationale behind the selection of this cellulose solvent as the reaction medium. As described by the complexation mechanism proposed by Burchard *et al.*, the extraction of protons at O-2 and O-3 (Scheme 1) drives the formation of a stable polyolatocopper complex cellulose under highly alkaline conditions.^38^ Accordingly, the polymeric chain units develop a negative charge. The generalized fabrication process for the preparation of PPy/BC composites is illustrated in Scheme 2. The procedure followed for the formation of PPy/NC composites was identical – except for the substitution of NC for BC. Initially, the BC material was introduced into the solvent system and stirred thoroughly to promote dispersion (or dissolution in cuoxam-based reaction media). This was followed by the addition of the pyrrole (Py) monomer solution and further mixing at room temperature. Sufficient time needed to be allotted to this step for maximal adsorption of Py onto the BC surface. The system was then cooled (to 0 °C) prior to dropwise addition of the FeCl_3_/HCl solution. Low temperatures and controlled addition of the oxidant/dopant solution encourage more orderly growth of PPy and reduce aggregation.^39^ As the reaction proceeded and PPy formed, the colorless mixture (aqueous reaction medium) transformed into a clear grey/black suspension of filamentous material. In contrast, the cuoxam-based solutions – which were initially dark blue/black in color – transformed into turbid brown suspensions. Acidification of the cellulose-cuoxam chelate facilitates regeneration of the dissolved cellulose. The dative covalent bonds (Cu–O-2 and Cu–O-3, Scheme 1) can be broken *via* protonation of the cellobiose units (*i.e.*, the addition of acid) to reprecipitate the cellulose fibers. Hence, addition of these suspensions to a concentrated HCl solution was necessary to quench the excess cuoxam and simultaneously regenerate the PPy-coated BC fibers. This transition was marked by the precipitation of black fibrous material in a clear yellow solution. This step was obviously unnecessary for composites prepared in aqueous reaction media, and thus omitted.

Finally, the composite suspensions were filtered *in vacuo* and washed with water, acetone and HCl. Water and acetone were used to remove contaminants (*e.g.*, unreacted Py or FeCl_3_). *In situ* p-doping (oxidation) of the PPy chains was achieved through the final washing step with HCl (dopant). Simultaneous diffusion of chloride anions (Cl^−^) into the polymer maintains electroneutrality.^40^ The delocalized positive charges (electron holes) enable charge transport between and along PPy chains to generate bulk conductivity.^41^ Since humidity can diminish performance, long-term storage of the paper-like PPy/BC composites in a desiccator was essential.

### Establishing morphological differences with TEM imaging

#### Effects of reaction media on morphology

**Fig. 1:**
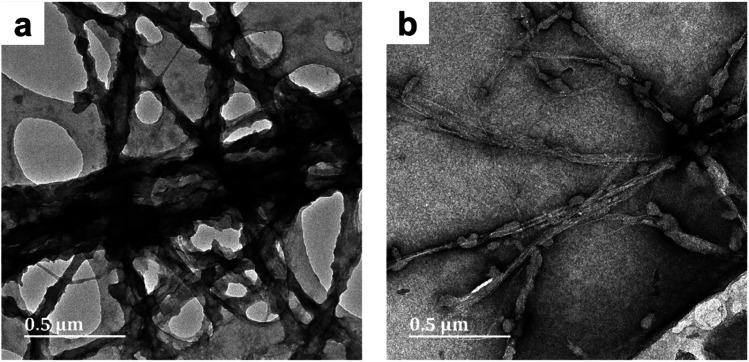
TEM micrographs of PPy/bacterial cellulose composites prepared in water (BC-A1, Fig. 1a) and low cuoxam concentrations (BC-B1; Fig. 1b), both with a 50 minute reaction duration.

The effect of reaction media on the morphology of the PPy/cellulose composites was studied by comparing the TEM micrographs obtained for BC-A1, BC-B1, BC-C1 and BC-D1. In BC-A1, a core-sheath morphology was observed – the core being BC fibers and the sheath being composed of fused PPy particles (Fig. 1a).

The increase in fiber diameter (*D*) from 40 nm (pristine BC) to approximately 78 nm (composite fibers) corresponds to a relatively uniform PPy coating with an average thickness of 19 nm. This result is in keeping with previous studies on the preparation of PPy/BC composites in an aqueous environment.^42,43^ In BC-B1, a sparse distribution of isolated PPy particles (*D* = 44 nm) on the BC template was observed (Fig. 1b). Hence, even the presence of minute quantities of cuoxam in the solvent system significantly affected the polymerization process. These findings were most likely caused by reduced adsorption of pyrrole monomers on the BC fibers due to a significant portion of these surface sites already being occupied by cuoxam – complexed with the cellulose backbone. Samples BC-C1 (Fig. S1a and b†) and BC-D1 (Fig. S1c and d†) both exhibited substantial inhomogeneity. Preparation of these samples occurred in highly concentrated cuoxam solutions. One would have expected that the improved dispersion of BC that occurs in this solvent environment would yield evenly coated and well separated PPy/BC fibers – as observed in other dispersing solvent systems.^36^ Instead, these samples featured dense aggregates of PPy accompanied by regions of largely uncoated or virtually pristine BC fibers. In these highly concentrated systems, the problems encountered with BC-B1 would most likely be further exacerbated. As the concentration of cuoxam increases, competition between Py monomers and cuoxam for access to the BC surface would likely intensify. Consequently, all of the Py monomers may end up distributed over a very limited (or at least reduced) surface area, resulting in the formation of dense aggregates upon polymerization. Concomitantly, once the solvent has been neutralized, the vast majority of the BC template would emerge in effectively pristine condition. Additionally, the strongly basic conditions associated with these solvent systems could have contributed to this morphology. Deprotonation of the –OH groups on both the BC and the Py monomers yields anionic derivatives. Coulombic repulsion between these species would have severely inhibited the adsorption of Py monomers – probably leading to the formation of unbound PPy aggregates. Following cellulose regeneration, adsorption of these aggregates onto the BC template is likely to have ensued.

#### Effects of reaction duration on morphology

**Fig. 2:**
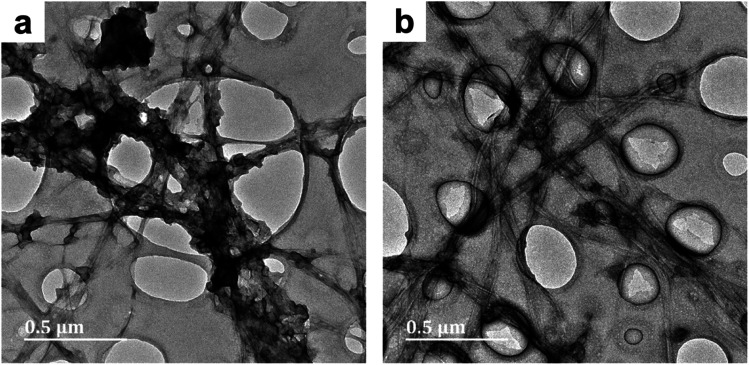
TEM micrographs of PPy/bacterial cellulose composite prepared in high cuoxam concentrations (BC-D2) with a 20 hours reaction duration. Both micrographs (a) and (b) belong to the different parts of the same composite (BC-D2).

The effect of reaction time was studied by comparing the sample series labelled 1 (50 minute reaction duration) with their equivalents (in terms of the solvent system) labelled 2 (20 hours reaction duration). Evaluating the micrographs of BC-D1 (Fig. S1c and d†) and BC-D2 (Fig. 2a and b), it became apparent that reaction duration affects the microstructure of the PPy deposits. After 50 minutes, a sparse distribution of aggregated PPy was observed. Meanwhile, after 20 hours a more uniform and even distribution of PPy on the BC template was observed. Thus, protracted polymerization appeared to improve the homogeneity of the PPy/BC composite. This result is supported by findings by Müller *et al.*^2^

#### Effects of template structure on morphology

**Fig. 3:**
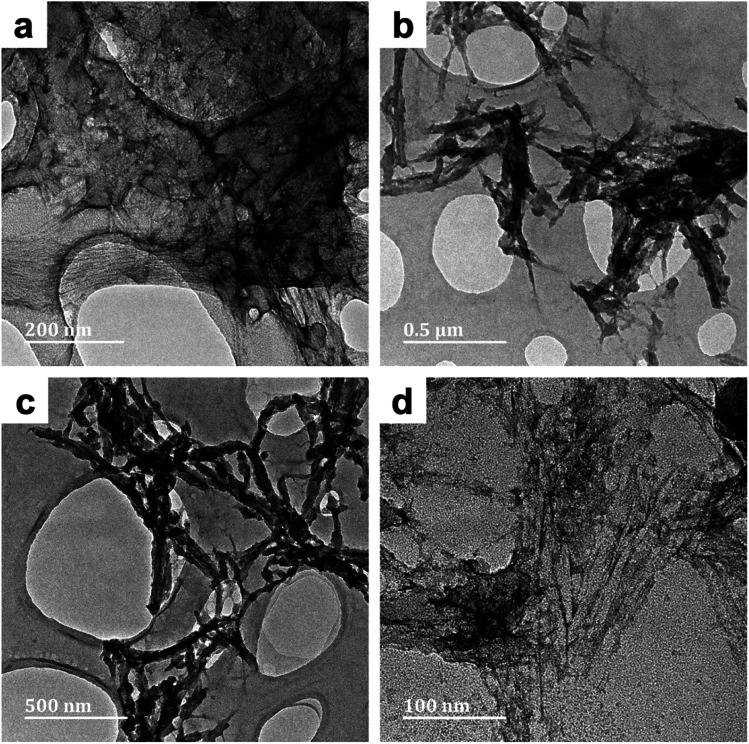
TEM micrographs of PPy/cellulose composites templated on bacterial cellulose (BC-A2; Fig. 3a) and nanocrystalline cellulose (NC-A2; Fig. 3b–d). Both composites were prepared in water with a 20 hours reaction duration.

The effect of structural variation of the cellulose template on the PPy/cellulose composites was studied by comparing samples: BC-A2 with NC-A2, and BC-C2 with NC-C2 (where bacterial cellulose- and nanocrystalline cellulose-templated samples are denoted BC and NC, respectively). The pristine BC sample existed as a ribbon-like network of long nanofibers, with an average diameter of 40 nm. By contrast the pristine NC sample, produced *via* sulfuric acid-catalyzed hydrolysis of the BC sample, was comprised of rod-like cellulose nanocrystals. The NC particles possessed an average diameter and length of 10 nm and 0.3 μm, respectively. TEM, FT-IR and XRD analysis of the cellulose templates is contained in the ESI.† In water, bacterial cellulose forms an entangled net-like structure, while unfunctionalised cellulose nanocrystals tend to agglomerate (especially at high concentrations). These discrepancies affect template accessibility for pyrrole adsorption during *in situ* polymerization. Solution behavior in cuoxam is discussed later. In sample BC-A2, deposition of PPy was evident, and the BC fibers appeared to be thoroughly entangled (Fig. 3a).

**Fig. 4:**
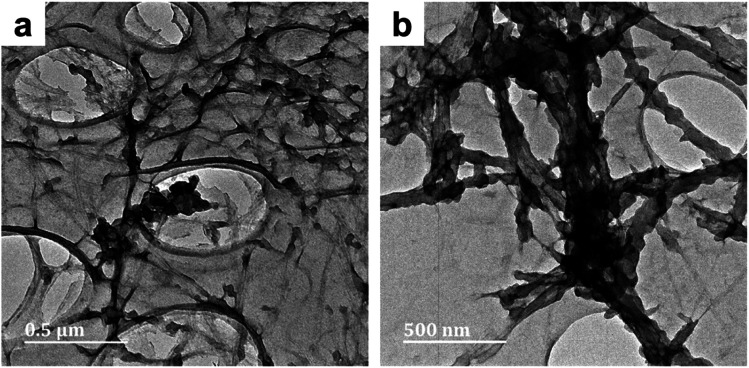
TEM micrographs of PPy/cellulose composites templated on bacterial cellulose (BC-C2; Fig. 4a) and nanocrystalline cellulose (NC-C2; Fig. 4b). Both composites were prepared in moderate cuoxam concentrations with a 20 hours reaction duration.

This could be due to additional hydrogen bonding interactions between proximate polypyrrole deposits – as well as nearby cellulose chains – leading to greater cohesion of the composite fibers. While in NC-A2, some nanofibres appeared to be uniformly coated with PPy, while others remained uncoated (Fig. 3b–d). Residual sulfate ester moieties on the NC surface improve the colloidal stability (*i.e.*, dispersibility) of these nanoparticles in aqueous solutions. This effect could have been responsible for the observed uniformity of the PPy coating of NC-A2. Nonetheless, great efforts were made to eliminate this surface functionality (0.20 at%S, Table S2†). Thus, localized aggregation of these nanoparticles *via* hydrogen bonds would still be conceivable, thereby limiting Py adsorption. This could account for the emergence of uncoated nanofibres. Samples BC-C2 and NC-C2 were prepared in concentrated cuoxam reaction media. A sparse distribution of PPy particles anchored on the surface of the BC network was observed for BC-C2 (Fig. 4a). A much more uniform and even coating of the NC fibers was observed for NC-C2 (Fig. 4b).

**Fig. 5:**
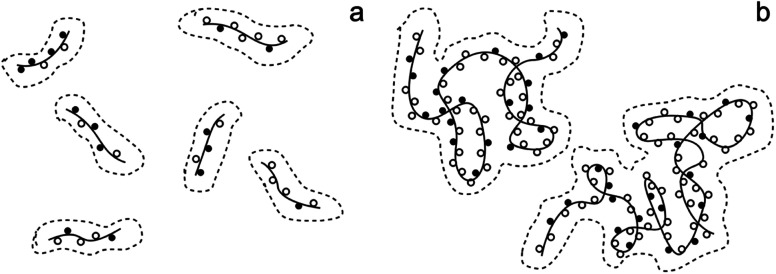
The proposed solution structure of cellulose in cuoxam: (a) short chains (DP_w_ < 1000) and (b) long chains (DP_w_ > 1000). The solid lines and the solid spheres represent cellulose chains and cuoxam-complexed anhydroglucose units, respectively. The hollow spheres signify imperfections in cuoxam binding. DP_w_ denotes the weight-average degree of polymerization. Adapted from Seger *et al.*^44^

The effect of molecular weight on polymer chain behavior in cuoxam could potentially explain these observations. Seger *et al.* described how intramolecular crosslinking and a reduction in chain stiffness (caused by imperfections in copper binding to cellulose segments) can lead to an entangled or coiled solution structure in high molecular weight samples (Fig. 5).^44^ Expression of this solution behavior could sterically hinder the adsorption of pyrrole onto the BC template. This phenomenon is not observed in low molecular weight cellulose which explains why the NC-templated sample featured a more homogeneous PPy coating.

#### Characterization using FT-IR and XRD

**Fig. 6:**
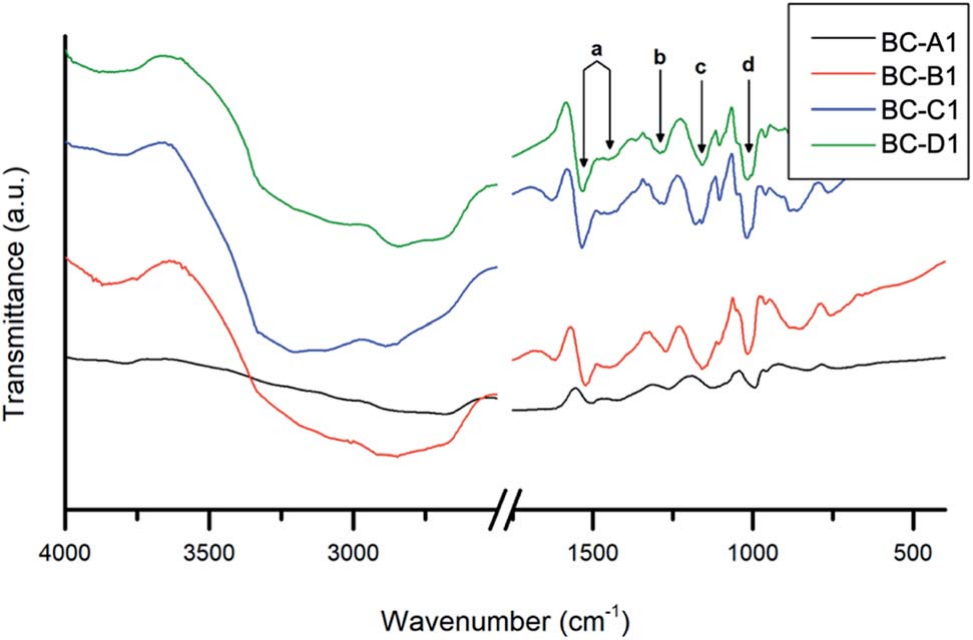
FT-IR spectra of the PPy/bacterial cellulose composites (series 1).

FT-IR spectroscopy is a useful tool for the characterization of surface bonding in materials. Additionally, covalent interactions between composite components can be evaluated. The spectra obtained for composite series 1 are presented in Fig. 6. Characteristic bands at 3342 cm^−1^ (O–H stretch) and 2915 cm^−1^ (asymmetric C–H stretch) are usually observed in a pure BC spectrum.^2^ Similarly, N–H stretching vibrations are responsible for a broad band at 3338 cm^−1^ in pure PPy spectra.^45^ In the composite spectra, these absorbance bands appeared as a single broad peak shifted to lower energies. This red-shift was likely due to hydrogen bonding interactions between the N–H and O–H moieties in cellulose and PPy, respectively.^46^

The composite absorbance patterns were largely comprised of overlapped absorbance bands contributed by each component. Nevertheless, a number of characteristic PPy bands (a–d) were apparent. The fact that these PPy peaks were well-defined in the composite spectra confirms that the polymerization of pyrrole was indeed accomplished.^47^ The peak assignment^2,48–50^ and measured energies of these absorbances are summarized in Table 2.

**Table 2:** Assignment of selected absorbance bands (a–d) from the FT-IR spectra of the PPy/bacterial cellulose composites (series 1)

	Wavenumber (cm^−1^)				Functional group assignment
	BC-A1	BC-B1	BC-C1	BC-D1	
a	1507, 1425	1522, 1452	1534, 1450	1532, 1450	C=C, C–N (stretching in pyrrole ring)
b	1261	1271	1290	1290	C–H (in-plane ring bending modes)
c	1123	1158	1160	1158	C–N (in-plane ring deformation and bending modes)
d	993	1052	1045	1047	C–H (bending modes)

Relative to the other samples, the spectrum obtained for BC-A1 was red-shifted. Furthermore, quenching of C–H (aliphatic) and O–H (hydroxyl) stretching vibrations, associated with cellulose, was evident. These findings suggest that the BC fibers in the BC-A1 sample were well-coated or insulated with a layer of PPy.^36^ This agrees with the previously discussed results of TEM analysis. The energy shift could be caused by a varying degree of doping between samples and the consequent influence of the counter-ion on bond strengths.^49,51^ Similar observations were made for series 2 (Fig. S3†). Notably, there was no discernible difference between the spectra of the NC-templated composites (NC-A2, NC-C2) and their BC-templated counterparts (BC-A2, BC-C2).

**Fig. 7:**
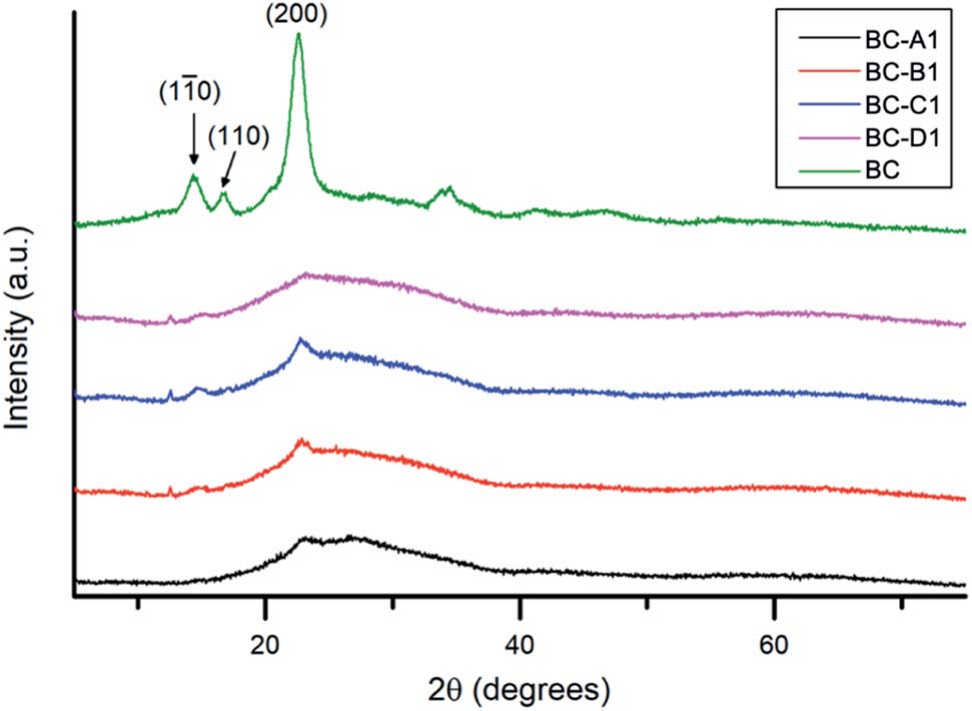
XRD spectra of the PPy/bacterial cellulose composites (series 1) and pristine bacterial cellulose (reference).

The XRD spectra obtained for series 1 and series 2 (along with pristine BC for reference) are presented in Fig. 7 and S4,† respectively. All of the diffraction peaks observed in the composite spectra could be indexed to characteristic peaks of cellulose I.^52^ The broad reflection around 15.2° was attributed to the overlap of (11̄0) and (110) reflections.^53^ The sharp shoulder peak at ∼22.5° corresponded to the (200) diffraction plane. The considerable background, in the range of 25–35° – observed only in the composite spectra – was likely caused by contributions from the broad diffraction peak of amorphous PPy.^54^ Compared with pristine BC, significant dampening of reflections was observed in the PPy/BC spectra. In keeping with previous findings, this indicates that the cellulose surface was at least partially coated with PPy.^45^

#### Effects of morphology on electrical conductivity

According to percolation theory, electrical conductivity is primarily dependent on the microstructure of the composites and their ability to form a conductive network.^55^ Improved connectivity is accompanied by a reduction in electrical resistance. This principle is central to the discussion that follows. The sheet resistance (*R*_s_) values of the composite films are reported in Table 3. Electrical conductivity (*σ*) is also reported for ease of comparison with literature.

**Table 3:** Sheet resistance and electrical conductivities of the PPy/bacterial cellulose composites

Sample	*R*_s_ (Ω sq^−1^)	*σ* (S cm^−1^)
BC-A1	(1.37 ± 0.33) × 10^2^	(1.94 ± 0.47) × 10^0^
BC-B1	(1.43 ± 0.15) × 10^3^	(1.76 ± 0.18) × 10^−1^
BC-C1	(3.61 ± 0.35) × 10^3^	(1.73 ± 0.89) × 10^−2^
BC-D1	(9.14 ± 3.28) × 10^5^	(1.26 ± 0.45) × 10^−3^
BC-A2	(3.66 ± 0.44) × 10^1^	(3.08 ± 0.37) × 10^0^
BC-B2	(4.73 ± 1.21) × 10^2^	(5.66 ± 1.45) × 10^−1^
BC-C2	(1.71 ± 0.44) × 10^3^	(2.08 ± 0.53) × 10^−1^
BC-D2	(4.33 ± 1.13) × 10^3^	(8.25 ± 2.15) × 10^−2^
NC-A2	(1.83 ± 0.23) × 10^2^	(1.39 ± 0.17) × 10^0^
NC-C2	(4.41 ± 1.59) × 10^2^	(2.61 ± 0.94) × 10^0^

These values were obtained by eqn S(3),† where *t* denotes the measured thickness of each sample. The electrical conductivities vary over a fairly narrow range in the order of 10^−3^ to 10^0^ S cm^−1^. The maximum conductivity was attained by BC-A2 (3.08 ± 0.37 S cm^−1^), a bacterial cellulose-templated composite prepared in aqueous reaction media for a duration of 20 hours. This compares well with the maximum of 7.34 S cm^−1^ obtained for similar PPy/BC composites prepared in DMF/H_2_O reaction media.^56^Table 4 presents a comparison of this work with similar conductive, nanocellulose-templated polypyrrole composites reported in the literature. Moreover, this composite afforded a roughly threefold increase in conductivity, when compared with pure PPy (1.14 S cm^−1^).^57^ This confirms the hypothesis that, given an aptly chosen template, the microstructure associated with templated PPy composites facilitates improved electron transport within the material. Meanwhile, BC-D1, the BC-templated composite prepared in the most concentrated cuoxam medium for a duration of 20 hours, yielded the poorest conductivity ((1.26 ± 0.45) × 10^−3^ S cm^−1^). Yet, this *s* value is comparable with that obtained for similar composite materials (1.4 × 10^−3^ S cm^−1^) that are purported to be sufficiently conductive for application in electrodes, electronic devices and sensors.^43^

**Table 4:** Synthesis and conductivity of various nanocellulose-templated polypyrrole composites reported in literature

Template^a^	Preparation method	Morphology	Conductivity (S cm^−1^)	Ref.
BC, NC	Oxidative polymerization in cuoxam solutions	See previous discussion	(1.26 × 10^−3^)-3.08	This work
BC	In situ oxidative chemical polymerization	Uniformly deposited spherical PPy nanoparticles (APS)	0.1–1.2	42
		Core–shell like structure (FeCl_3_)		
BC	In situ chemical polymerization	3-D network of nanofibres coated with PPy ‘nanosheath’	7.34	56
BC	Oxidative polymerization in DMF-H2O solutions	Core-sheath nanofibres	∼77	36

a NC and BC denote nanocrystalline and bacterial cellulose, respectively.

**Fig. 8:**
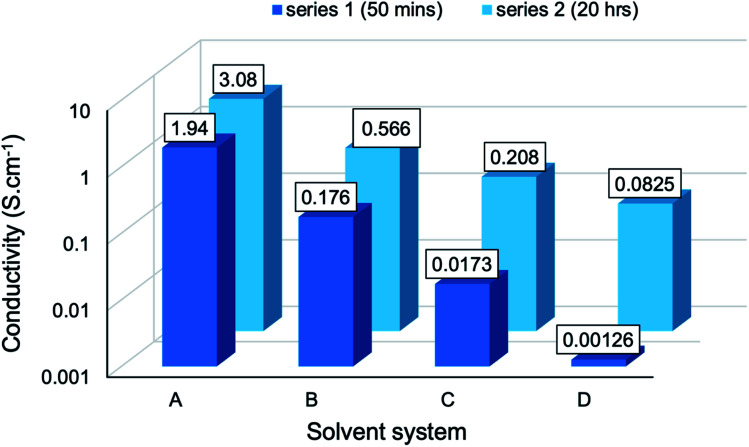
Conductivity of the PPy/bacterial cellulose composite films (series 1 and 2) prepared in different solvent systems. A denotes water; B, C and D denote low, moderate, and high cuoxam concentrations, respectively.

The conductivity of composites from series 1 and series 2 have been presented graphically (Fig. 8) to establish the effect of reaction media. A clear trend emerged: as the solvent environment was modified from an aqueous (A) to a highly concentrated cuoxam solution (D), the conductivity decreased significantly from values of order 10^0^ to 10^−3^ S cm^−1^. This trend can be attributed to the morphological differences elucidated by TEM analysis. As previously discussed, the introduction of a minute quantity of cuoxam elicited a substantial transformation from a relatively uniform (BC-A1) to a sparse distribution (BC-B1) of PPy particles on the BC network (Fig. 1). Cellulose itself is considered to be a non-conductive material (insulator). Consequently, the sparsely distributed PPy particles are separated by this insulating material, thereby inhibiting the formation of conductive pathways – which would inevitably impede electron flow. Hence, the observed decrease in *s* values from BC-A1 to BC-B1. The further increase observed for BC-C1 and BC-D1 could also be rationalized by comparing composite morphology (Fig. S1†). In these samples, dense aggregates of PPy were found.

These aggregates serve as localized regions with excellent conductivity. Nevertheless, these few conductive hotspots were insulated by the BC fiber matrix which remained virtually uncoated – and hence non-conductive. Thus, macroscopically, electrical conductivity was very poor and the *s* values became very small. In BC-D1, this problem was exacerbated (due to an even higher concentration of cuoxam) which accounts for the further decrease in conductivity. It is also apparent that series 1 exhibited higher *s* values than series 2. TEM analysis revealed that the increase in reaction duration from 50 minutes (X) to 20 hours (Y) improved the homogeneity of the PPy coating. It follows that a more even distribution of PPy throughout the composite would lead to a greater density of conductive pathways, ultimately improving bulk conductance.

**Fig. 9:**
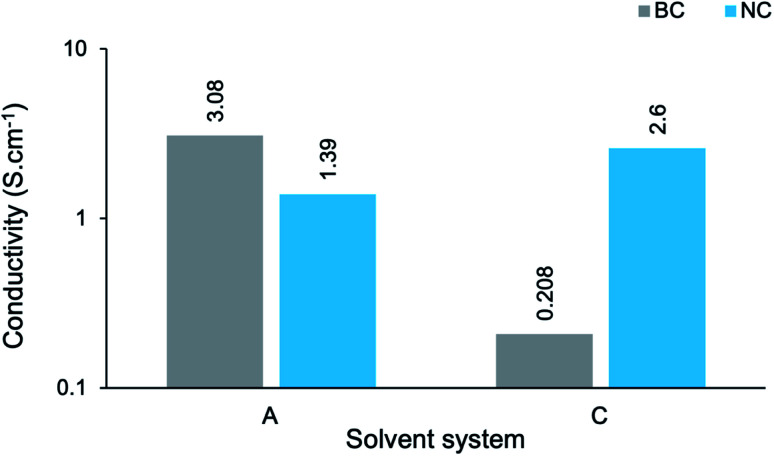
Conductivity of PPy/cellulose composite films templated on bacterial cellulose (BC-A2 and BC-C2) and nanocrystalline cellulose (NC-A2 and NC-C2), prepared in different solvent systems with a 20 hours reaction duration. A denotes water; C denotes moderate cuoxam concentrations.

In order to establish the impact of the dimensionality (or aspect ratio) of the cellulose template on conductivity, the *s* measurements of BC-A2, NC-A2, BC-C2 and NC-C2 have been presented graphically (Fig. 9). In an aqueous environment (A), the BC-templated composite possessed a higher *s* value, albeit relatively similar in magnitude, than the NC-templated composite. Meanwhile, in a moderately concentrated cuoxam solution (C), the opposite was found to be true. Once again, microstructural variations can be used to rationalize these results. Although NC-A2 displayed more uniform coating of the cellulose template than BC-A2, the presence of pristine NC fibers could impede the conduction of electrons due to their insulating nature (Fig. 3a). Furthermore, the NC sample was isolated as short rod-like particles (length ≪ 300 nm). In contrast, ultrafine BC fibers formed an entangled network. It is easy to comprehend how the intrinsic connectivity of BC would improve conductance. From previous arguments, the dense, even polymer coating of NC (NC-C2) can easily rationalize the disparity in *s* values between BC-C2 and NC-C2 (Fig. 9).

#### Effects of morphology on surface wettability

The contact angle (CA) values obtained for the composite films are reported in Table 5. For clarification, all reported measurements refer to the water contact angle. Except for NC-A2 (nanocrystalline cellulose-templated composite prepared in water; 20 hours reaction duration), all of the composites displayed a contact angle less than 90°. Hence, the PPy/cellulose composites tended towards hydrophilic behavior – with the best wetting behavior (CA = 23.09 ± 1.08°) exhibited by BC-D1 (bacterial cellulose-templated composite prepared in high cuoxam concentrations; 50 minute reaction duration).

**Table 5:** Contact angle measurements of the PPy/cellulose composites

Sample	Contact angle (degrees)
BC-A1	35.78 ± 2.22
BC-B1	57.60 ± 1.67
BC-C1	58.30 ± 3.72
BC-D1	23.09 ± 1.08
BC-A2	48.48 ± 4.25
BC-B2	65.92 ± 4.54
BC-C2	23.19 ± 1.79
BC-D2	25.75 ± 0.01
NC-A2	101.64 ± 2.12
NC-C2	61.18 ± 2.87

**Fig. 10:**
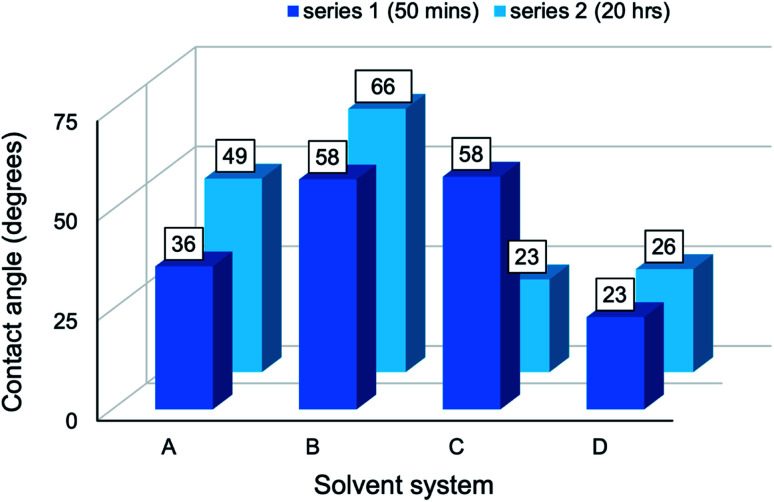
Contact angle measurements of the PPy/bacterial cellulose composite films (series 1 and 2) prepared in different solvent systems. A denotes water; B, C and D denote low, moderate, and high cuoxam concentrations, respectively.

The CA measurements of composites from series 1 and series 2 have been presented graphically (Fig. 10) to establish the effect of reaction media on wettability. A common trend was apparent: as the solvent environment changed from an aqueous (A) to a highly concentrated cuoxam solution (D), the CA increased initially and then gradually began to decline. A maximum was reached in system B (lowest cuoxam concentration of B-D) for both the series 1 and 2. Additionally, the minimum CA was attained at higher cuoxam concentrations (C or D), suggesting that very high concentrations of cuoxam diminished the CA to lower values than those initially obtained for composites prepared in aqueous media. By implication, highly concentrated cuoxam reaction media can be implemented in the design of PPy/cellulose composite materials with improved surface wettability (or hydrophilicity). This could provide new opportunities for these materials in biomedical, filtration, and anti-fogging applications.^58^

**Fig. 11:**
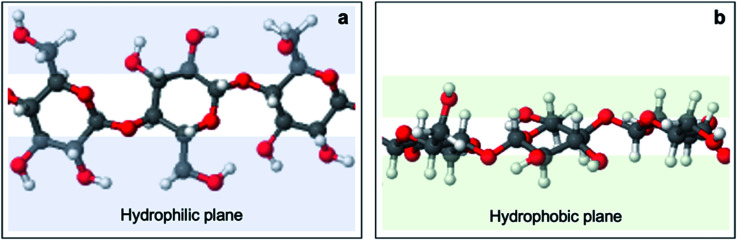
Anisotropic structure of cellulose I. Red, white, and grey spheres represent oxygen, hydrogen and carbon atoms, respectively. An overhead view (a) shows that equatorial positions are occupied by hydroxyl groups, forming hydrophilic planes (blue) at the sides of the anhydroglucose unit ring plane. A side view (b) shows that axial positions are occupied by aliphatic protons, forming hydrophobic planes (green) above and below the anhydroglucose unit ring plane. Adapted from Yamane *et al.*^59^

To rationalize this behavior, one needs to look at the effect of the structural anisotropy of cellulose on wettability, studied by Yamane *et al.*^59^ Cellulose is constituted of anhydroglucose units (AGUs) which adopt a chair conformation. The axial positions are occupied by hydrogen atoms of C–H bonds, which creates hydrophobicity in the axial direction. In contrast, AGUs are hydrophilic in the equatorial direction since all three hydroxyl groups are equatorially bonded (Fig. 11). In the unit cell of regenerate cellulose, the AGUs are orientated such that the plane on which these rings lie is perpendicular to the (11̄0) crystallographic planes. Hence, the surface of the (11̄0) plane is associated with a high density of equatorially positioned hydroxyl groups, creating an exceptionally hydrophilic surface.

**Fig. 12:**
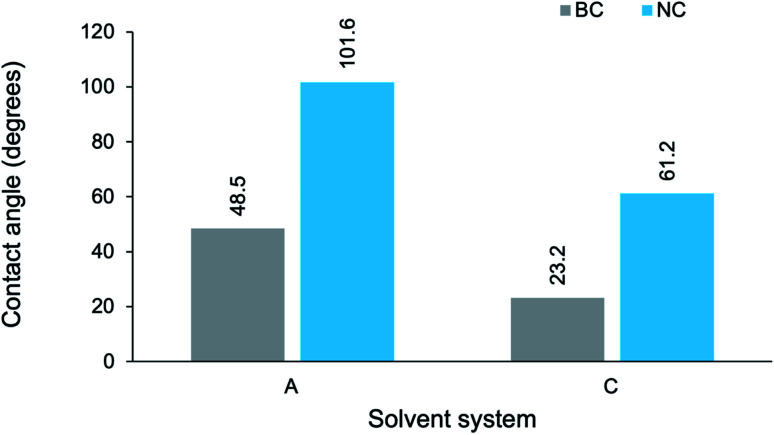
Contact angle measurements of PPy/cellulose composite films templated on bacterial cellulose (BC-A2 and BC-C2) and nanocrystalline cellulose (NC-A2 and NC-C2), prepared in different solvent systems with a 20 hours reaction duration. A denotes water; C denotes moderate cuoxam concentrations.

Additionally, experiments have shown that the (11̄0) planes constitute a large fraction of the surface area of regenerated cellulose films.^59^ It follows that regenerated cellulose films exhibit extraordinary wetting (*i.e.* low CAs). This could explain the observed decrease of CA measurements in increasingly concentrated cuoxam solutions. A higher cuoxam concentration enables the dissolution of a larger fraction of BC, and hence a higher fraction of regenerated cellulose would be present in the final film. The consequent improvement in film hydrophilicity is concomitant with the measurement of a reduced CA. Doping effects can potentially explain the disparity in measured contact angles for composites prepared in system A and system B. TEM analysis revealed that solvent A induced a relatively uniform and even coating of PPy particles on the BC network (BC-A1, Fig. 1a). A sparse distribution was evident in solvent B (BC-B1, Fig. 1b). Therefore, given the greater quantity of PPy present, one would also expect more counterions (Cl^−^) from the dopant to be adsorbed on the surface of composites prepared in solvent A. Since the dopant ions are hydrophilic, improved wettability should result. This could explain why lower contact angles for BC-A1 and BC-A2 were observed. Series 1 and 2, corresponding to a reaction duration of 50 minutes and 20 hours, respectively, displayed the same trend with similar magnitudes. Therefore, it seems that reaction duration did not play a significant role in wetting behavior. In order to study the influence of the dimensionality (or aspect ratio) of the cellulose template on wettability, the contact angle (CA) measurements of BC-A2, NC-A2, BC-C2 and NC-C2 have been presented graphically (Fig. 12).

It is apparent that the NC-templated composites exhibited significantly higher contact angles (at least twice as large) than the BC-templated composites. Moreover, in the case of BC-A2 (48.5°) and NC-A2 (101.6°), a marked shift from hydrophilic to hydrophobic wetting behavior was observed. TEM analysis revealed that, relative to their BC-templated counterparts, the NC-templated composites possessed a more uniform coating of PPy nanoparticles. This can translate into reduced droplet contact area, as well as composites with reduced droplet permeability.^7,60^ Hence, one can rationalize the significantly higher contact angles measured for the NC-templated composites (NC-A2 and NC-C2). By implication, control over the morphology of the cellulose template enables manipulation of the wetting properties displayed by the resulting composite. Furthermore, an array of composites which span a broad range of contact angles – encompassing both wetting and non-wetting behaviors – can be produced *via* manipulation of this experimental parameter. The reason for the general decline in CA observed from system A to system C has been discussed.

## Conclusions

A series of polypyrrole/cellulose composites were prepared in cuoxam (or Schweizer's reagent) solutions of varying concentration. A detailed account of the synthesis and characterization of these composites is presented. This study investigated the effect of reaction media and cellulose template morphology on electrical conductivity and surface wetting. In general, the templated polypyrrole composites demonstrated hydrophilic behavior – with the exception of nanocrystalline cellulose-templated composites, exhibiting a bias towards non-wetting behavior. Hence, microstructural variation of the cellulose template serves as a facile approach to tailor composite surface wettability. Substitution of aqueous reaction media with highly concentrated cuoxam solutions elicited a pronounced improvement in the wetting of the composites, while low cuoxam concentrations improved hydrophobicity. Hence, this work highlights the ability of Schweizer's reagent to control the wettability of conductive cellulose composites. The composites exhibited reasonably good conductivities (maximum of 3.1 S cm^−1^), with several composites showing a response superior to pristine polypyrrole. Increasingly concentrated cuoxam reaction media yielded composites with progressively inferior conductivities. This contradicts the initial hypothesis which stated that preparation in cuoxam would favor the adoption of homogeneous morphologies (uniformly polypyrrole-coated cellulose) with improved electroconductivity. However, this study does indicate that composite microstructure, wettability, and conductivity are highly sensitive to the cuoxam concentrations in reaction media and, hence, to the extent of cellulose dissolution – hinting at the potential application of cellulose solvents as an economical and facile means to fine-tune the morphology and properties of *in situ* functionalized cellulose nanocomposites. Further work exploring other types of cellulose solvents to form structurally and functionally distinct composites is underway.

## Author contributions

The manuscript was written through contributions of all authors. All authors have given approval to the final version of the manuscript.

## Conflicts of interest

There are no conflicts to declare.

## Supplementary Material

RA-012-D2RA04320C-s001
