# Supplementary material for: In situ polymerization and electrical conductivity of polypyrrole/cellulose nanocomposites using Schweizer's reagent
Source: RSC Adv. 2022 Aug 9;12(34):22031–43. doi: 10.1039/d2ra04320c (PMC9361926; doi:10.1039/d2ra04320c)
Supplement: RA-012-D2RA04320C-s001 [file RA-012-D2RA04320C-s001.pdf]

Electronic Supplementary Material (ESI) for RSC Advances.
This journal is © The Royal Society of Chemistry 2022

ELECTRONIC SUPPLEMENTARY INFORMATION

In situ polymerization and electrical conductivity of polypyrrole/cellulose nanocomposites using Schweizer's reagent

*Stephanie A. Fraser and Werner E. van Zyl**

School of Chemistry and Physics, University of KwaZulu-Natal, Westville Campus, Durban, 4000, South Africa.

**Corresponding author: vanzylw@ukzn.ac.za*

S1. Thickness measurements of PPy/cellulose composites.	2
S2. Mechanism of pyrrole polymerization.	2
S3. TEM images of PPy/cellulose composites.	3
S4. FT-IR and XRD spectra of PPy/cellulose composites.	4
S5. Synthesis of Schweizer's reagent.	5
S6. Experimental details for preparation and characterization of cellulose.	6–9
S7. TEM, FT-IR and XRD analysis of cellulose.	10–13
S8. STEM-EDS analysis of PPy/cellulose composites.	14-18

Composite thickness measurements

Table S1. Thickness measurements of the PPy/cellulose composites.

Sample	Thickness (mm)
**BC-A1**	0.04
**BC-B1**	0.04
**BC-C1**	0.03
**BC-D1**	0.01
**BC-A2**	0.09
**NC-A2**	0.04
**BC-B2**	0.04
**BC-C2**	0.03
**NC-C2**	0.01
**BC-D2**	0.03

Pyrrole polymerization mechanism

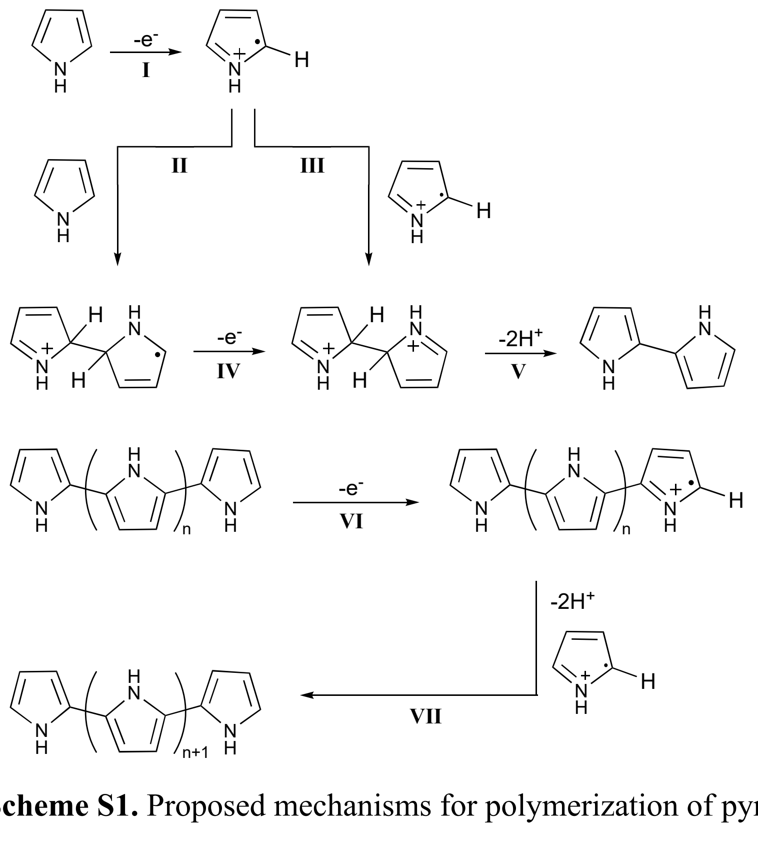

Scheme S1. Proposed mechanisms for polymerization of pyrrole (adapted from Tan *et al.*[1]).

Supplementary TEM imaging results (PPy/cellulose composites)

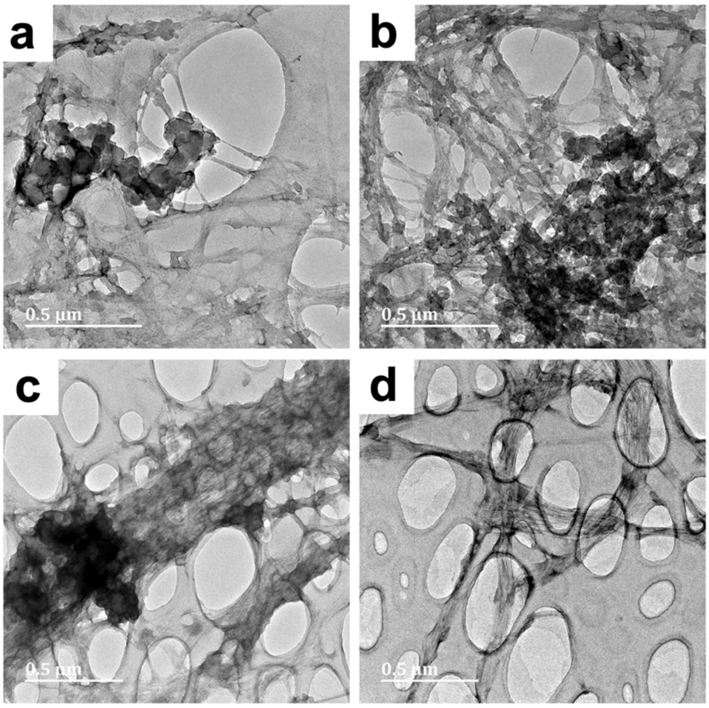

Figure S1. TEM micrographs of PPy/bacterial cellulose composites prepared in moderate (**BC-C1**, Figure S1a and S1b) and high (**BC-D1**; Figure S1c and S1d) cuoxam concentrations, both with a 50-minute reaction duration.

Supplementary FT-IR and XRD results (PPy/cellulose composites)

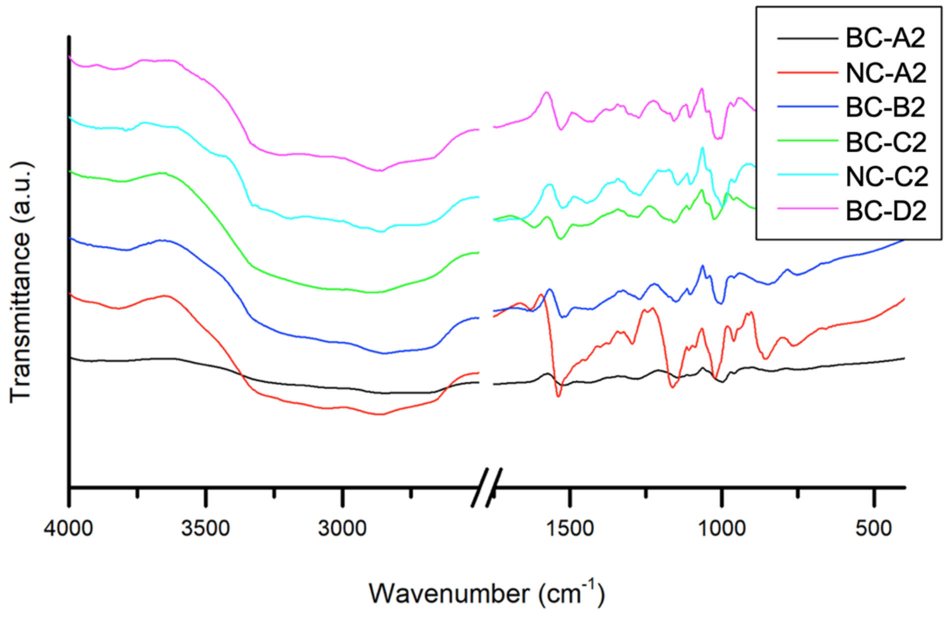

Figure S2. FT-IR spectra of the PPy/cellulose composites (series **2**).

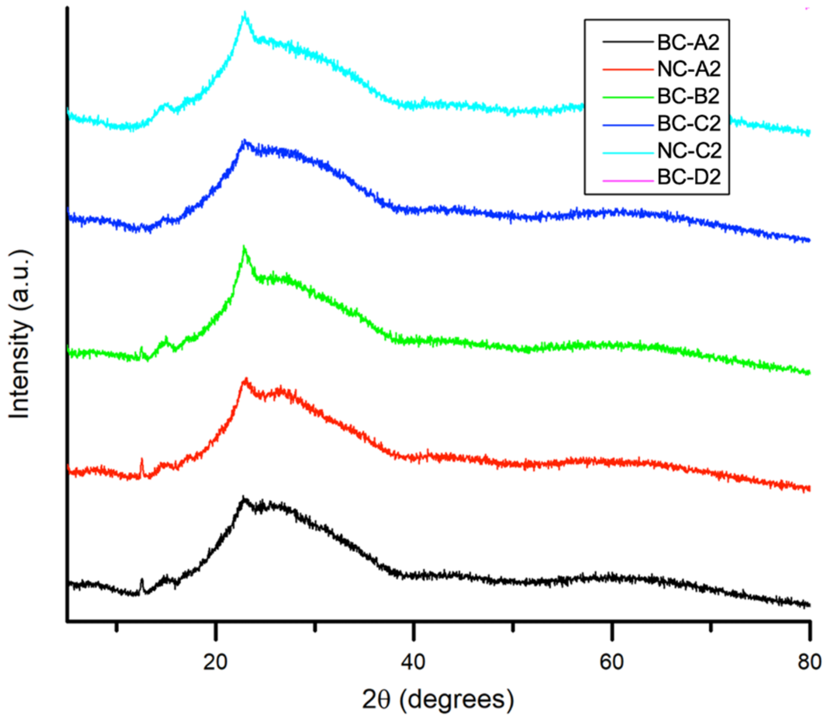

Figure S3. XRD spectra of the PPy/cellulose composites (series **2**).

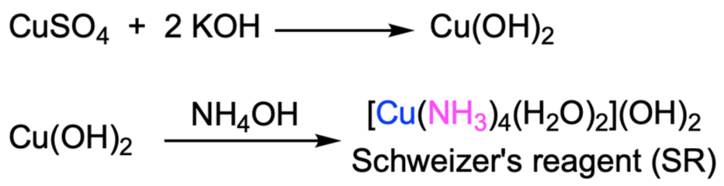

Scheme S2. Synthesis of Schweizer’s reagent (or cuoxam).

Supplementary experimental details (cellulose samples)

Synthesis of bacterial cellulose (BC): There were three main stages involved in the preparation of bacterial cellulose. First, a sufficiently established Symbiotic Culture of Bacteria and Yeast (SCOBY) was cultivated. This was followed by the preparation of the growth medium, which was subsequently inoculated with the SCOBY to initiate the biosynthesis of BC. Finally, chemical treatment and mechanical processing of the BC pellicle formed in the growth medium yielded a suspension of purified BC. The process of BC preparation is summarized in Scheme S3.

Cultivation of a symbiotic culture of bacteria and yeast (SCOBY): In a 5 L plastic beaker, white sugar (98.95 g) was dissolved in boiling water (840 mL). To the sugar-water mixture, two green tea bags (~6 g) were added and steeped for four minutes. A volume of water (480 mL) was then added to the beaker, and the solution was left to cool to 25 °C. The teabags were then removed and the sweet tea solution was inoculated with commercially sourced kombucha tea (240 mL). The final solution was then evenly distributed between several glass beakers. Each beaker was covered with cheesecloth held in place by a rubber band. Fermentation was carried out in a dark cupboard at room temperature (~25 °C) for approximately three weeks (or until the pellicles were ≥1 cm in thickness).

Preparation of the bacterial cellulose (BC) growth medium: To prepare a 1.2 L batch of the growth medium, two green teabags (~6 g) were added to boiling water (1 L) and subsequently brewed for approximately 20 minutes. While still warm, white sugar (200 g) was added and dissolved in the tea solution. Once the sweet tea solution had cooled to a temperature of 30 °C, vinegar (200 mL) was added.

Preparation of pure bacterial cellulose (BC): The prepared growth medium (6 L) was poured into a sterilized plastic tray, and one of the pre-prepared SCOBY pellicles was transferred to the growth medium. The tray was then covered with a cloth and incubated at room temperature (~25 °C) in a dark and static environment. After two to three weeks, a thick pellicle of cellulose was removed from the tray and thoroughly rinsed with deionized water. The cellulose was sliced into large pieces and placed in a

1 L glass beaker. A solution, prepared by combining deionized water (250 mL) and 15% sodium hypochlorite solution (25 mL), was added and the solution was stirred for three days to sterilize the cellulose. The cellulose was then removed and thoroughly rinsed in deionized water. Thereafter, a 0.25 M NaOH (200 mL, 50.0 mmol) solution was prepared in a 1 L glass beaker. After being stirred in this solution for 24 hours, the cellulose was removed and rinsed with deionized water. Finally, the cellulose was mechanically disintegrated using a kitchen blender (Bosch SilentMixx Blender, 700 W) to form a thick, homogeneous suspension.

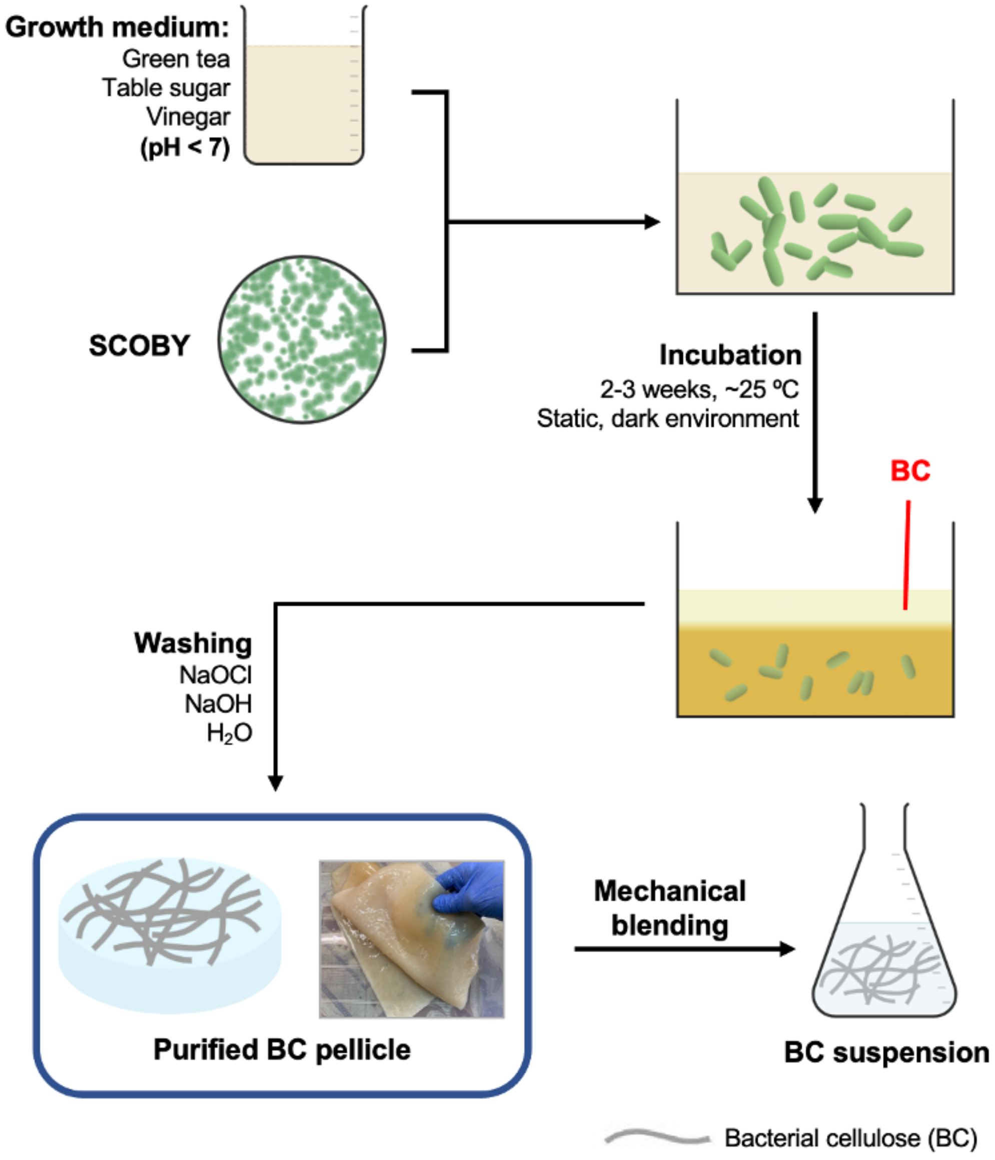

Scheme S3. Preparation of bacterial nanocellulose.

Synthesis of nanocellulose (NC): A portion of a 0.7 wt.% BC suspension was transferred to a 200 mL glass beaker. An appropriate volume of 60% (w/w) H_2SO_4 solution was added (10 mL H_2SO_4 : 1 g cellulose (dry mass)). The solution was heated to 60 °C and the temperature was maintained for 70 minutes, with vigorous stirring. Once sufficiently hydrolyzed, the solution was quenched with an excess of cold water (4 °C). Thereafter, the cellulose suspension was transferred to dialysis bags (cellulose membrane, MWCO: 14 kDa) and dialyzed against deionized water for seven days (or until the suspension reached a stable pH). The dialysate was replenished twice daily. Subsequently, the NC suspension was centrifuged at 3000 rpm for 90 minutes using the Grant-bio Laboratory Centrifuge instrument (LMC-3000). Nanocellulose suspensions were prepared by diluting the cellulose pellet with an appropriate volume of deionized water followed by sonication. The suspensions were stored in glass containers at room temperature.

Supplementary characterization details (cellulose samples)*

*XRD and FT-IR measurements were obtained as previously described in the Experimental Section of the manuscript.

XRD analysis: Average crystallite size (D) was evaluated for three characteristic reflections (of cellulose I) using the Scherrer equation, Equation S1, where K denotes the Scherrer constant (or shape factor), λ is the wavelength of incident X-ray radiation, β is the peak broadening at half the maximum height (FWHM) of the instrument-corrected line profile in radians, and θ is the Bragg diffraction angle.[2] Crystallinity index (C.I.) was evaluated using the semi-empirical Segal method, shown in Equation S2, where I_{cr}, corresponding to the crystalline contribution, denotes the height of the dominant diffraction peak between 2θ = 22-23°; and I_{am}, corresponding to the amorphous contribution, denotes the minimum peak intensity between 2θ = 18-19°.[3]

TEM imaging of cellulose samples: A small volume of each cellulose suspension was transferred onto a copper grid and left to dry for approximately ten minutes under a heat lamp. All samples were stained with a drop of uranyl acetate (1% w/v solution) to enable

visualization of cellulosic material. TEM images were captured using a JEOL 2100 high-resolution transmission electron microscope (HRTEM) operated at 20 kV.

Equations

$$D = \frac{K\lambda}{\beta \cos \theta} \quad \text{(S1)}$$

$$C.I. = \frac{I_{cr} - I_{am}}{I_{cr}} \times 100 \quad \text{(S2)}$$

$$\sigma = \frac{1}{R_s \times t} \quad \text{(S3)}$$

References

1 Y. Tan and K. Ghandi, *Synth. Met.*, 2013, **175**, 183–191.

2 P. Scherrer, in *Kolloidchemie Ein Lehrbuch*, ed. R. Zsigmondy, Springer Berlin Heidelberg, Berlin, Heidelberg, 1912, pp. 387–409.

3 L. Segal, J. J. Creely, A. E. Martin and C. M. Conrad, *Text. Res. J.*, 1959, **29**, 786–794.

Supplementary results (cellulose samples)

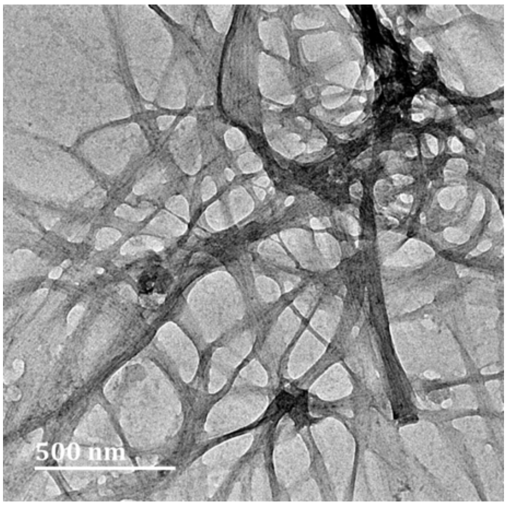

Figure S4. TEM micrograph of BC.

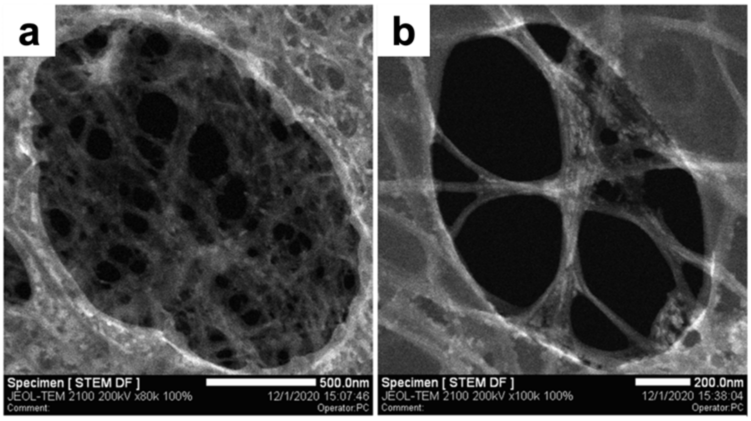

Figure S5. STEM darkfield micrographs of (Figure S5a) BC and (Figure S5b) NC.

Table S2 Elemental composition of BC and NC.

	Atomic percent (%)*		
	C	O	S
BC	70.44	19.88	-
NC	92.92	4.23	0.20

*Obtained from STEM-EDX analysis of STEM darkfield micrographs (Figure S5)

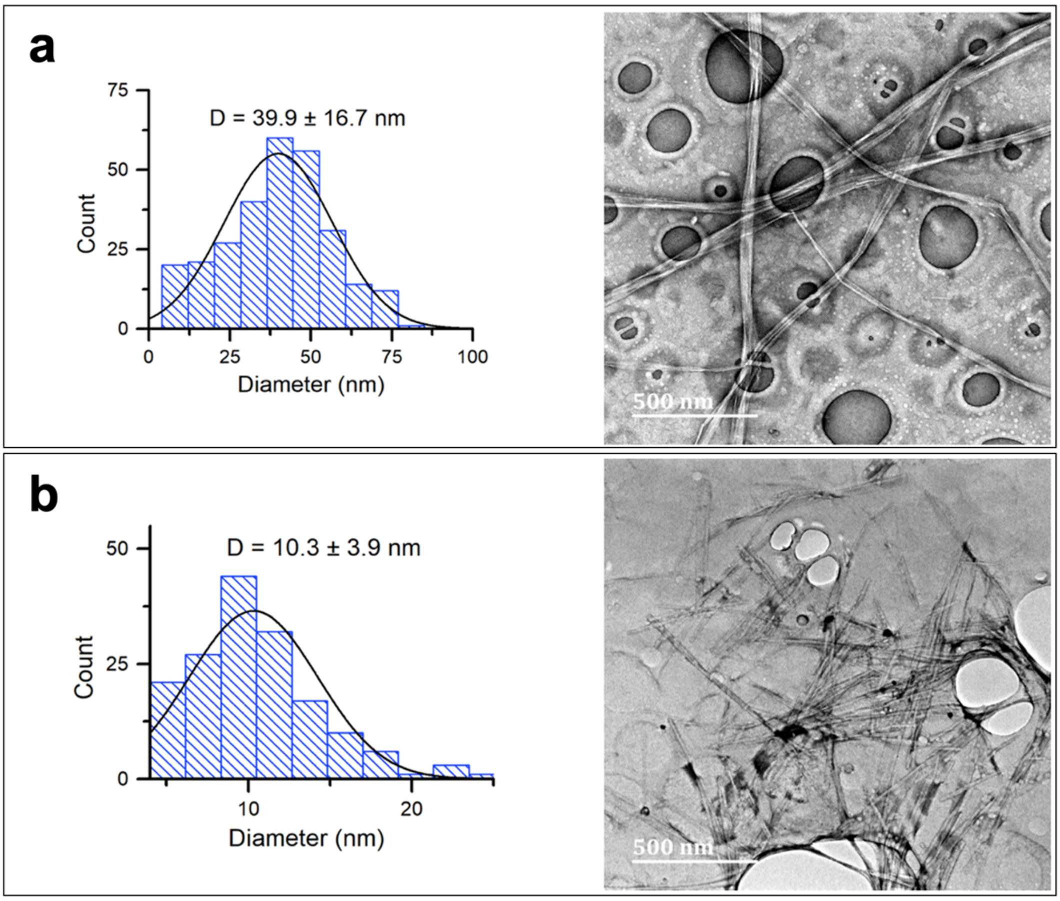

Figure S6. Transmission electron micrographs and corresponding histograms showing the diameter (D) size distribution for (Figure S6a) BC and (Figure S6b) NC.

Table S4. The dimensionality of cellulose samples.

	Length (nm) †	Diameter (nm) †	Aspect ratio
BC	n/a‡	39.9 ± 16.7	n/a‡
NC	317 ± 136	10.3 ± 3.9	30.8 ± 24.9

†Dimensions of cellulose nanostructures were determined from TEM micrographs.
‡Exact length (and hence aspect ratio) could not be determined at the high magnifications used in TEM.

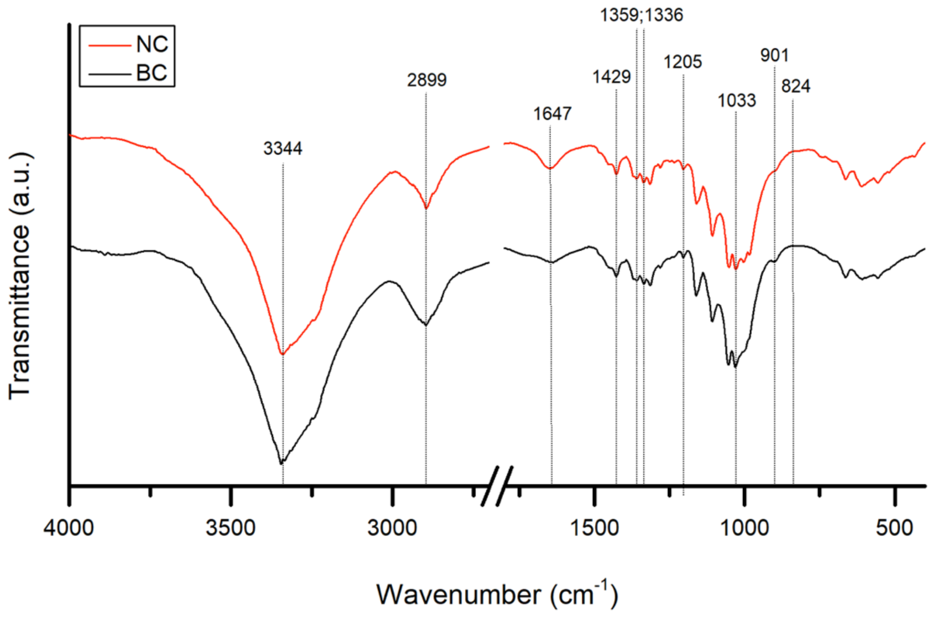

Figure S7. Fourier transform infrared (FT-IR) spectra of cellulose samples.

Table S3. Peak assignment of the FT-IR spectra of cellulose samples.

Wavenumber (cm^{-1})	Functional group assignment
3344	O-H (stretching)
2899	C-H (stretching)
1647	absorbed water
1429<br>1359<br>1336<br>1033<br>901	-CH2, -CH, -OH, C-O (bending & stretching)
824	β-glycosidic bond (vibration)

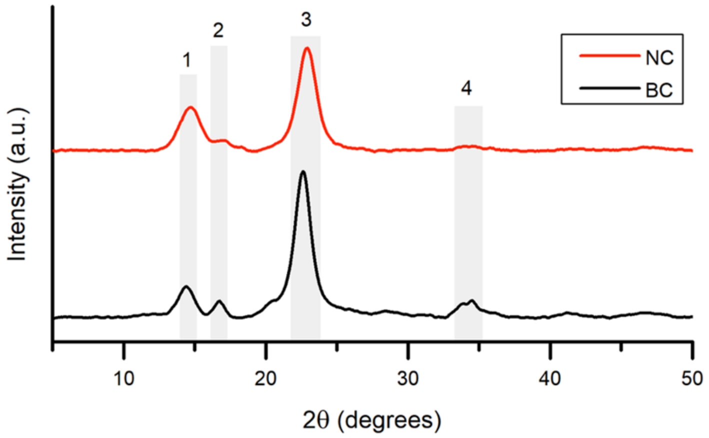

Figure S8. X-ray diffraction (XRD) spectra of cellulose samples.

Table S5. The crystallite size and crystallinity index of cellulose samples.

	Crystallite size (nm)†			CI (%)†
	D_1	D_2	D_3	
BC	6.70	11.03	5.48	72.5
NC	5.11	6.22	5.17	64.8

†Crystallite size and CI were determined from the X-ray diffractograms (Figure S8).

STEM-EDS analysis of PPy/cellulose composites

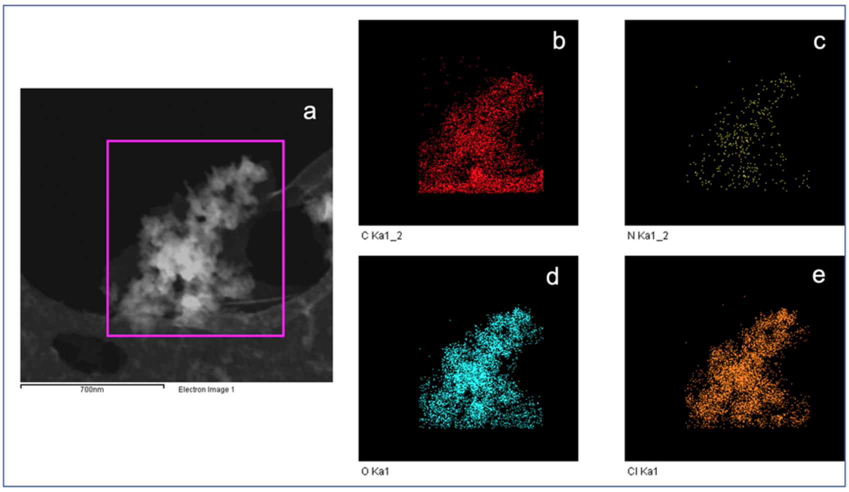

Figure S9. STEM-EDS mapping of composite BC-A1: a) micrograph of sample and elemental distribution of b) carbon, c) nitrogen, d) oxygen and e) chlorine.

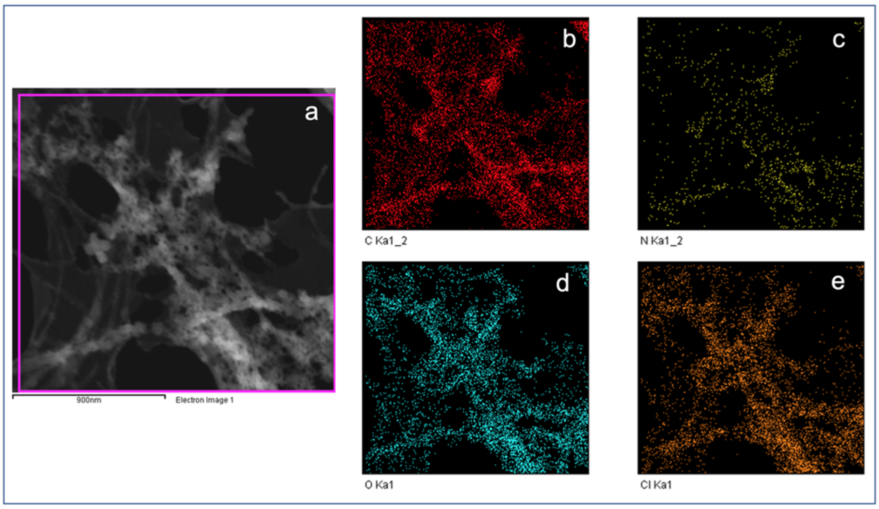

Figure S10. STEM-EDS mapping of composite BC-B1: a) micrograph of sample and elemental distribution of b) carbon, c) nitrogen, d) oxygen and e) chlorine.

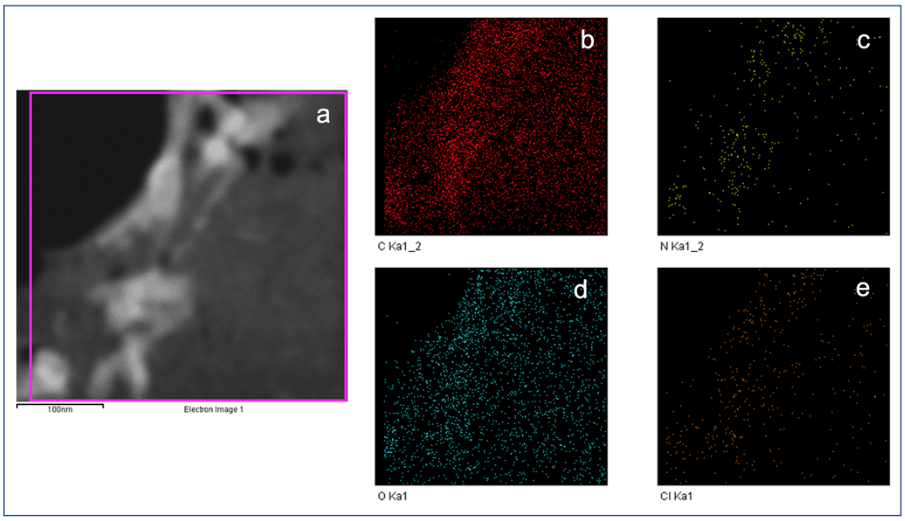

Figure S11. STEM-EDS mapping of composite BC-C1: a) micrograph of sample and elemental distribution of b) carbon, c) nitrogen, d) oxygen and e) chlorine.

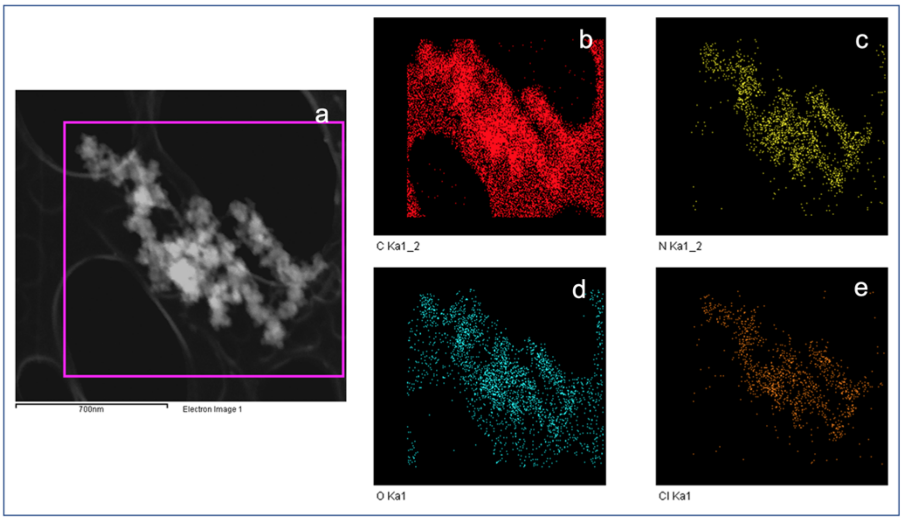

Figure S12. STEM-EDS mapping of composite BC-D1: a) micrograph of sample and elemental distribution of b) carbon, c) nitrogen, d) oxygen and e) chlorine.

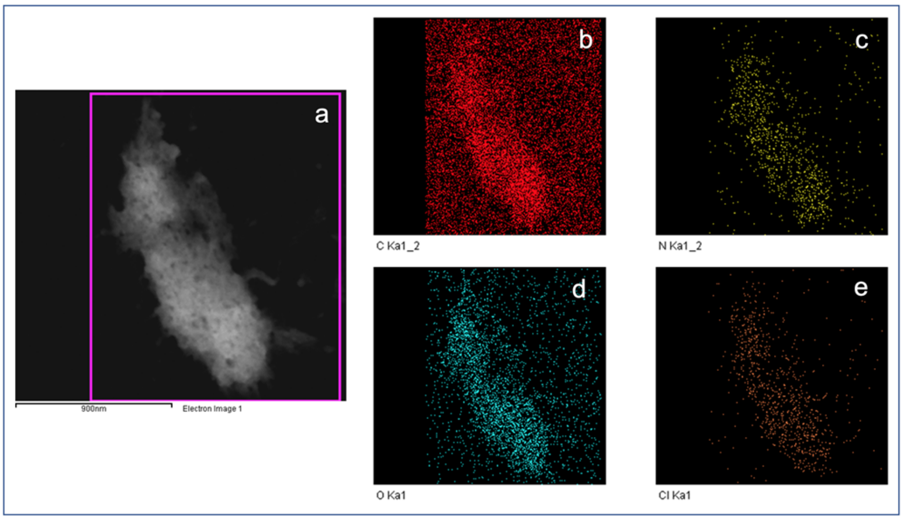

Figure S13. STEM-EDS mapping of composite BC-A2: a) micrograph of sample and elemental distribution of b) carbon, c) nitrogen, d) oxygen and e) chlorine.

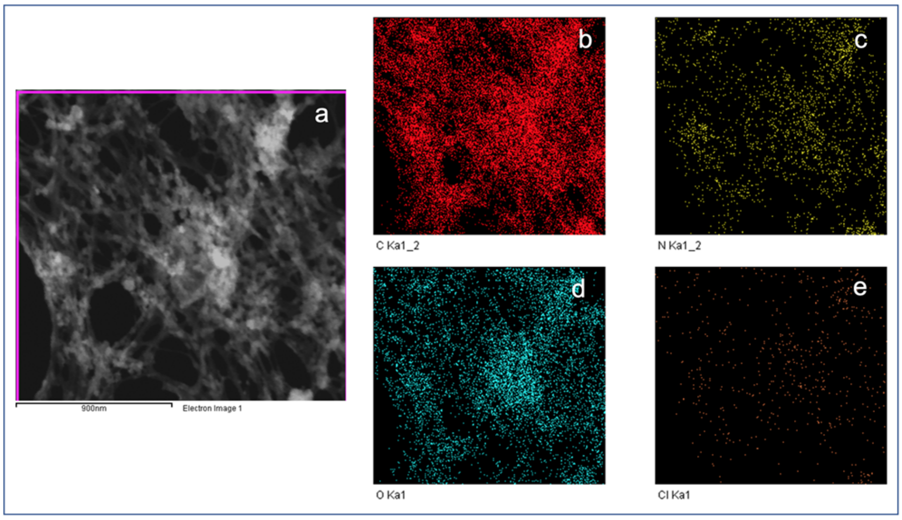

Figure S14. STEM-EDS mapping of composite BC-B2: a) micrograph of sample and elemental distribution of b) carbon, c) nitrogen, d) oxygen and e) chlorine.

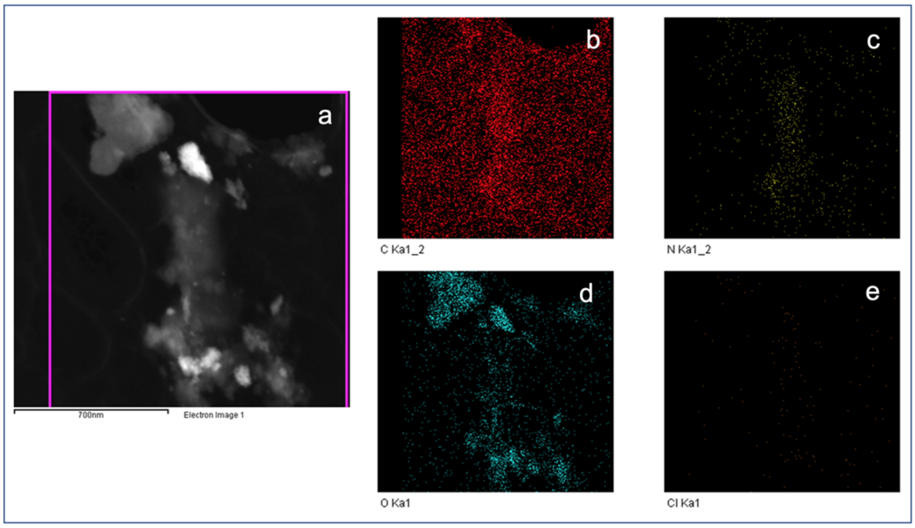

Figure S15. STEM-EDS mapping of composite BC-C2: a) micrograph of sample and elemental distribution of b) carbon, c) nitrogen, d) oxygen and e) chlorine.

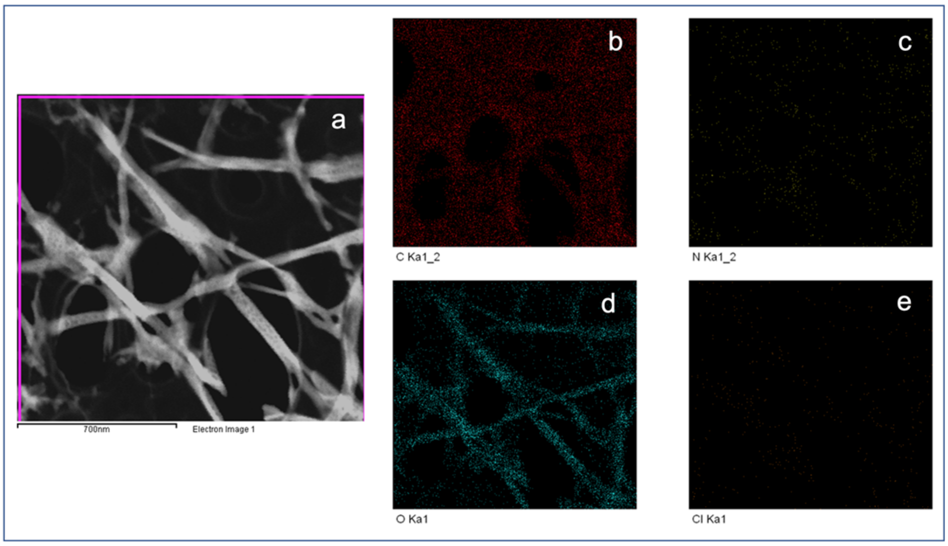

Figure S16. STEM-EDS mapping of composite BC-D2: a) micrograph of sample and elemental distribution of b) carbon, c) nitrogen, d) oxygen and e) chlorine.

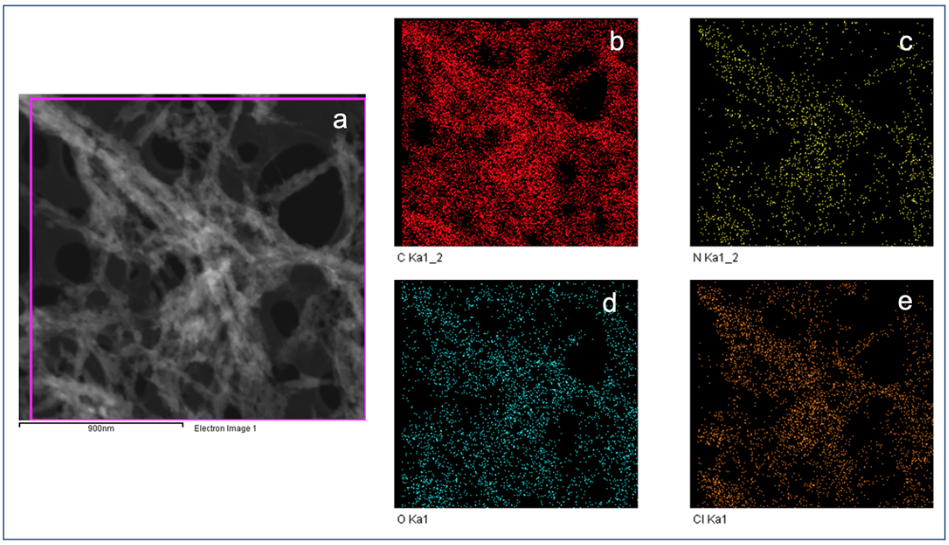

Figure S17. STEM-EDS mapping of composite NC-A2: a) micrograph of sample and elemental distribution of b) carbon, c) nitrogen, d) oxygen and e) chlorine.

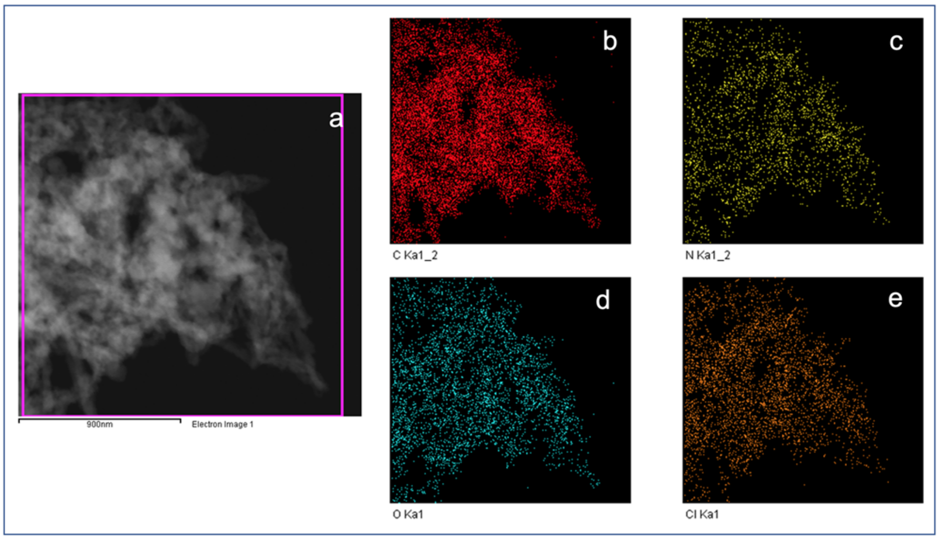

Figure S18. STEM-EDS mapping of composite NC-C2: a) micrograph of sample and elemental distribution of b) carbon, c) nitrogen, d) oxygen and e) chlorine.
